# Heat stress upregulates arachidonic acid to trigger autophagy in sertoli cells via dysfunctional mitochondrial respiratory chain function

**DOI:** 10.1186/s12967-024-05182-y

**Published:** 2024-05-26

**Authors:** Yu Hu, Nan Jian Luo, Lu Gan, Hong Yan Xue, Ke Yan Luo, Jiao Jiao Zhang, Xian Zhong Wang

**Affiliations:** 1 Department of Reproductive Medicine, Department of Obstetrics and Gynecology, Affiliated Hospital of Zunyi Medical University, Affiliated Hospital of Zunyi Medical University, 563000 Zunyi, China; 2 Chongqing Key Laboratory of Forage and Herbivore, College of Veterinary Medicine, Southwest University, 400715 Chongqing, Beibei China; 3 Department of Preclinical Medicine, Zunyi Medical University, 563000 Zunyi, China

**Keywords:** Heat stress, Arachidonic acid, Autophagy, Mitochondrial stress

## Abstract

As a key factor in determining testis size and sperm number, sertoli cells (SCs) play a crucial role in male infertility. Heat stress (HS) reduces SCs counts, negatively impacting nutrient transport and supply to germ cells, and leading to spermatogenesis failure in humans and animals. However, how HS affects the number of SCs remains unclear. We hypothesized that changes in SC metabolism contribute to the adverse effects of HS. In this study, we first observed an upregulation of arachidonic acid (AA), an unsaturated fatty acid after HS exposure by LC-MS/MS metabolome detection. By increasing ROS levels, expression of KEAP1 and NRF2 proteins as well as LC3 and LAMP2, 100 µM AA induced autophagy in SCs by activating oxidative stress (OS). We observed adverse effects of AA on mitochondria under HS with a decrease of mitochondrial number and an increase of mitochondrial membrane potential (MMP). We also found that AA alternated the oxygen transport and absorption function of mitochondria by increasing glycolysis flux and decreasing oxygen consumption rate as well as the expression of mitochondrial electron transport chain (ETC) proteins Complex I, II, V. However, pretreatment with 5 mM NAC (ROS inhibitor) and 2 µM Rotenone (mitochondrial ETC inhibitor) reversed the autophagy induced by AA. In summary, AA modulates autophagy in SCs during HS by disrupting mitochondrial ETC function, inferring that the release of AA is a switch-like response, and providing insight into the underlying mechanism of high temperatures causing male infertility.

**Supplementary Information:**

The online version contains supplementary material available at 10.1186/s12967-024-05182-y.

## Introduction

Heat stress (HS) has been known to provoke reproductive system disorders and spermatogenesis issues in humans and mammals [1]. High ambient temperature negatively affects testicles, resulting in cellular damage, as maintaining the low temperature for the testicles (approx. 2–7 °C below core body temperature) in summer poses a significant challenge [2–4]. It is therefore urgent for clinicians to address the problem related to male infertility due to the decline of good quality semen during the summer months. Sertoli cells (SCs) play a crucial role in spermatogenesis by establishing the blood-testis barrier, and providing nutrients and structural support to germ cells [5]. The population of SCs during the development of testis not only determines germ cell numbers and the ultimate testicular size, but also affects the overall output of sperm production [6, 7]. Thus, the loss or damage to SCs can lead to impaired spermatogenesis and even permanent infertility [8].

Mounting evidence indicates that the exposure to HS induces apoptosis and autophagy in both germ cells and somatic cells [2, 9, 10]. The autophagic process play a vital role in maintaining homeostasis, promoting cell differentiation, cell development, and cell survival by eliminating molecules and subcellular components via lysosome-mediated degradation [11]. Proteins LC3, Beclin-1 and P62 are central autophagy components involved in autophagy flux [12]. LC3 is a mammalian homolog of the yeast ATG8 protein, an ubiquitin-like protein in autophagosome membranes that becomes lipidated. LAMP2, a lysosomal-associated membrane protein, directly correlates with LC3-positive autophagosomes [13]. P62 which can promote LC3-I accumulation is required for the formation of LC3-I positive structures in autophagy-deficient cells [14]. Recently study highlighted that autophagy initiation occurs at mitochondria membrane coupling site [15]. Dysfunctional mitochondria were associated with mitochondrial electron transport chain (ETC) and lead to oxidative stress (OS) [16]. Autophagy boosts tumor cell metabolism by supplying metabolic substrates such as glucose and amino acids to maintain mitochondrial functions. In response to energy stress, autophagy can react to depletion of cellular ATP levels, as seen acute inhibition of the mitochondrial respiratory chain or glucose deprivation, as well as to changes in cytoplasmic Ca^2+^ and reactive oxygen species(ROS) [17, 18]. Moreover, ETC deficiency inhibits autophagy lysosomal hydrolysis either by activating AMPK signaling or by directly activating the lysosomal Ca^2+^ channel [18]. Although previous studies have demonstrated that mitochondria activate the autophagic pathways to alleviate OS in human cells and yeast via Diacylglycerol kinase and All-trans-retinal [19, 20], it is unclear which metabolite regulates mitochondrial autophagy under HS in SCs.

Arachidonic acid (AA), a ω-6 polyunsaturated fatty acid, is present in phospholipids of cell membranes. The increase of AA concentrations under stress can change the function and structure of the biofilm, increasing its permeability and brittleness [21]. Dall and Faergeman reported that ω-3 polyunsaturated fatty acids (PUFAs) triggered autophagy which kill cancer cells, while ω-6 PUFAs-mediated autophagy extended the lifespan of Caenorhabditis elegans or human epithelial cells, indicating that PUFAs can modulate autophagy [22]. Lei showed that AA metabolites regulated the apoptosis of β-cell involving in the activation of endoplasmic reticulum Ca^2+^-channel, the activity of mitochondrial ATPase as well as phospholipase A2 (PLA2, an enzyme involved in the release of AA) [23]. However, the mechanism of AA affect autophagy is unknown. Here, we hypothesized that AA could induce autophagy by altering mitochondrial ETC function during HS in SCs.

As pigs and humans share many anatomical and physiological characteristics, pigs are expected to be an ideal tissue and organ source for xenotransplantation, regenerative medicine, animal models of human hereditary diseases, or animal bioreactors of recombinant human proteins or biopharmaceuticals. Therefore, pig testicular SCs were used in this study. All of the piglets’ testicles were characterized at the same period to avoid sex disparities and the impact of distinct sex estrus cycles on metabolic performance. We first identified the significant increase of AA under HS by LC-MS/MS analysis. Additionally, we evaluated the role of mitochondrial complex-ROS axis that regulates AA inducing autophagy. As a result, this study helps to elucidate the underlying mechanisms that regulate autophagy during AA exposure, providing a new perspective on male infertility caused by HS, as well as contributing to the understanding of the clinical impact of small molecule metabolites.

## Methods and materials

### Chemical reagents and antibodies

Stock inhibitors were diluted in dimethyl sulfoxide (DMSO) prior to use. The final working concentrations of reagents were as follows: AAT (arachidonic acid, Cat# HY-109590, 100 µM), AAY (arachidonic acid metabolism inhibitor, Diethylcarbamazine citrate, Cat# HY-12642, 100 µM), NAC (N-Acetyl Cysteine, 5 mM), Rotenone (150 nM). The following primary antibodies were used in Western Blot analysis and Immunofluorescence analysis: Complex I, II, and IV (oxphos) (Thermo Fisher, Cat# 45-8099), KEAP1 and NRF2 (CST, Cat# 8047 and #12721), Lamin β1, LC3 and P62/SQSTM1 (Proteintech, Cat# 12987-1-AP, 18725-1-AP, 18420-1-AP), Alexa Fluor 488 Anti-Mouse /Rabbit IgG (Elabscience, Cat# E-AB-1056), Alexa Fluor 594 Anti-Mouse/Rabbit IgG (Elabscience, Cat# E-AB-1060).

### Primary SCs culture

The SCs were isolated from boar testes and purified as previously described [24]. Briefly, capsulate and spermatic cord of testes were removed before cutting into small pieces, followed by digesting with 0.3% type IV collagenase for 40 min and 0.25% trypsin for 20 min at 32 °C shaker (70 rpm). In this experiment, cells were centrifugated (1500 g, 5 min), followed by filtering by 0.180 mm and 0.0374 mm mesh sieves. Subsequently, cells were resuspended in 1:1 DMEM/Ham’s F12 medium (Gibco, Cat# 11,559,716), 1% Penicillin-Streptomycin Solution (Solarbio, Cat# P1400), 10% FBS (Gibco, Cat# 10099-141) and 1% glutamine (Gibco, Cat# 25,030,081) and loaded into 6-well cell culture plates at a density of 1.6 × 105 cells/mL in 32 °C, 5% CO2 incubator to form a confluent monolayer. Purified SCs were homogenized and cultured under the HS treatment (44 °C for 1 h), normal treatment (32 °C for 1 h), AAT/AAY treatment (100 µM for 6 h), NAC treatment (5 mM for 6 h), Rotenone (150 nM for 6 h) before protein extraction.

### LC-MS/MS analysis

#### Metabolite extraction

The collected samples (each group consisting of 8 replicates) were thawed on ice, and metabolites were extracted using a 50% methanol buffer. Briefly, 20 µL of sample was mixed with 120 µL of precooled 50% methanol, vortexed for 1 min, and incubated at RT for 10 min; the extraction mixture was subswquently stored overnight at -20 °C. After centrifugation at 4,000 g for 20 min, supernatants were transferred into new 96-well plates. These samples were stored at -80 °C until LC-MS analysis. To ensure data quality, pooled samples (quality control, QC) were prepared by combining 10 µL of each extraction mixture.

### Liquid oxidation treatment

All samples were acquired by the LC-MS system according to the machine protocol. Firstly, all chromatographic separations were performed using an ultra-performance liquid chromatography (UPLC) system (SCIEX, UK). An ACQUITY UPLC BEH Amide column (100 mm*2.1 mm, 1.7 μm, Waters, UK) was used for the reversed phase separation. The column oven was maintained at 35 °C. The flow rate was at 0.4 mL/mins and the mobile phase consisted of solvent A (25 mM ammonium acetate + 25 mM NH3·H2O) and solvent B (IPA: ACN = 9:1 + 0.1% formic acid). Gradient elution conditions were set as follows: 0∼0.5 min, 95% B; 0.5∼9.5 min, 95–65% B; 9.5∼10.5 min, 65∼40% B; 10.5∼12 min, 40% B; 12∼12.2 min, 40∼95%B; 12.2∼15 min, 95% B. Each sample was injected with a volume of 4 µL.

### Mass spectrum treatment

Metabolites eluted from the column was detected using A high-resolution tandem mass spectrometer TripleTOF5600plus (SCIEX, UK). The Q-TOF was operated in both positive and negative ion modes, with following parameters: the curtain gas at 30 PSI, Ion source gas1 at 60 PSI, Ion source gas2 at 60 PSI, and an interface heater temperature at 650 °C. The Ionspray voltage floating was set at 5000 V and − 4500 V in positive ion mode and negative ion mode, respectively. The mass spectrometry data were acquired in IDA mode. The TOF mass ranges from 60 to 1200 Da. The survey scans were acquired in 150 ms and up to 12 product ion scans were collected if the intensity exceeds a threshold of 100 counts per second (counts/s) with a 1 + charge-state. Total cycle time was fixed to 0.56 s. Four time bins were summed for each scan at a pulser frequency value of 11 kHz through monitoring of the 40 GHz multichannel TDC detector with four-anode/channel detection. Dynamic exclusion was set at 4 s. During the acquisition, the mass accuracy was calibrated every 20 samples. Furthermore, to evaluate the stability of the LC-MS during the entire acquisition, a quality control sample (Pool of all samples) was acquired after every 10 samples.

### **Cell viability and toxicity determination**

Cell viability was evaluated by the CCK-8 Kit according to the manufacturer’s instructions (Beyotime, Cat# C0037). Briefly, SCs were aliquoted into 96-well plates at a density of 0.6 × 104 cells per well, and cultivated at 32 °C in a humidified atmosphere with 5% of CO2 until reaching approximately 70% confluence of cells. Treatment of cells with indicated treatment followed by addition of fresh culture media with 10 µL CCK-8 solution to each well, followed by incubating for 2 h. The absorbance was detected through the Microplate Reader (Bio-Rad, USA) at 450 nm to assess the viability of SCs. Toxicity of cells was evaluated through the content of lactate dehydrogenase (LDH) using LDH reagent kit (NJJCBIO, Cat# A020-2-2) following the instructions. The absorbance at 490 nm was measured using a Microplate Reader (Bio-Rad, USA).

### Detection of MDA and ROS

Cells were crushed by an ultrasonic shaker prior to the detection of MDA, ROS and Ca^2+^. MDA was measured using the Lipid Peroxidation (MDA) Assay Kit (Abcam, Cat# ab118970). MDA is converted into MDA-TBA adduct by reaction with TBA in the sample. The conversion can then be measured calorimetrically at 532 nm. The intracellular ROS content was determined using ROS Detection Reagents (Invitrogen, Cat# D399). Following cells reaching at 100% confluence in black 96-well dark plates, ROS detection was carried out using the indicated treatments. Cells were then incubated with 25 µM H2DCFDA probes indicators in warm 1×PBS at 32 °C incubator for 30 min. Subsequently, ROS content was measured using a spectrophotometer at an excitation wavelength of 485 nm and an emission wavelength of 530 nm.

### Western blot and immunofluorescence

A bicinchoninic acid assay (BCA) protein assay kit (Bioground, Cat# BG0020) was used to determine the total protein concentration in SCs lysates lysed with cell lysis buffer (Thermo Fisher, Cat# 89,900). The denatured protein was electrophoresed on a polyacrylamide gel for 8–12% SDS-polyacrylamide and then being transferred onto a PVDF membrane (Roche, Cat# 3,010,040,001). Blots were blocked in 5% bovine serum albumin (BSA) and incubated in primary antibodies at a dilution of 1:1000 overnight at 4 °C, followed by an incubation with anti-rabbit horseradish peroxidase-conjugated secondary antibodies (1:1500; Beyotime, China). Bots were detected using SuperSignal® West Pico Chemiluminescent Substrate (Millipore, Cat# WBKlS0100) on a chemiluminescent imager (Bio-Rad, USA) and quantified using Image Lab Software (Bio-Rad, USA). Densitometry data were normalized to Lamin β1 expression. For immunofluorescence, cells at 100% confluence were exposed to the indicated treatments, then fixed, blocked, and stained as previously described [14]. Briefly, cells were fixed with 4% paraformaldehyde (PFA) at RT for 10∼15 min, followed by a neutralization with 100 mM glycine. Cells were blocked in 5% BSA blocking buffer and incubated overnight at 4 °C with primary antibody diluted at 1: 500 followed by incubation with secondary antibody (1:1000) and DAPI (1:1000) at RT for 1 h. The fluorescence signals were visualized using Olympus imaging microscope.

### Co-immunoprecipitation

According to the previous description [25], cells were washed in pre-chilled PBS before adding cold RIPA lysis buffer (1 mL per 10^7^ cells). After centrifugation at 14,000 g at 4 °C for 15 min, the supernatant was immediately transferred into new tubes. Protein A/G-agarose beads were washed twice in PBS, then a 6% protein A/G agarose working solution was prepared by adding 50 g of protein A/G agarose for each mL of sample solution. To eliminate non-specific binding proteins, the mixture of samples were shaken on a horizontal shaker for 10 min at 4 °C. Then, the protein A/G-agranule beads was removed post the centrifugation at 14,000 g at 4 °C for 15 min and the supernatant was collected. Finally, total protein using the BCA assay was quantify and proteins that bind to each other were identified using western blot analysis.

### Transmission electron microscopy (TEM)

1 × 10^6^ of SCs were seeded in 25 cm^2^ flask cultures reaching 100% confluency and then treated as indicated. Cells were trypsinized, pelleted, washed, and fixed in 2.5% glutaraldehyde for 30 min at 4 °C. The cells were washed three times followed by centrifuging at 1000 rpm for 5 min each time, and then fixed in 1% osmium tetroxide at RT for 10 min. The cells were dehydrated in increasing concentrations of ethanol (70, 85, 95, 100%) for 1 h. Then the cells were infiltrated with a 1∶1 mixture of propylene oxide, and Epon at RT for 1 h, and then placed in Pure Epon overnight. The samples were cured at 40 °C for 24 h and then cured at 60 °C for 48 h prior to section. Imaging was performed using Haptic H-7100 transmission electron microscopes (Hitachi, Japan).

### Mitochondrial energy metabolism and mitochondrial membrane potential (MMP) detections

The Seahorse XF Cell (Agilent, XFe24) was used to measure the oxygen consumption rate (OCR) and extracellular acidification rate (ECAR) for mitochondria. The mitochondria were isolated using Percoll density gradient centrifugation as previously described [26]. DMEM or RPMI medium supplemented with Seahorse XF containing 1 mM pyruvate, 2 mM glutamine, and 10 mM glucose was used for the assay medium. The protein concentration of isolated mitochondria was measured. An equal amount of mitochondria (1 g protein/well) was plated in the Seahorse cell culture microplate. OCR and ECAR measurements were conducted sequentially following the baseline measurements as followed. The mitochondrial ORC function was evaluated with the following solutions: (A) oligomycin (2.5 µg/mL final), (B) FCCP (4 µM final), and (C) antimycin A (40 µM final) + rotenone (2 µM final) [27]. ECAR was assessed in the glucose, oligomycin, FCCP and 2-deoxy-glucose (2-DG) before incubating the plate at 37 °C for 30 min and analyzing.

Mitochondrial membrane potential (MMP, ΔΨm) was measured using flow cytometry probes. JC-1 (Cayman, Cat# 701,560) dye was used as an indicator of mitochondrial membrane potential. Fluorescence was measured at λex = 488 nm and λem = 530–615 nm. In normal SCs cells, the cells were stained with orange-red fluorescence.

### Statistical analyses

SPSS 29.0 (Version 29.0; SPSS Inc, Chicago, IL, USA) was used for data statistics and analysis of variance. The Homogeneity analysis was performed otherwise the arc-sine transformation was applied. An unpaired t-test was utilized to compare SC metabolism data between the HS and NC groups. A one-way analysis of variance was performed to assess the effect of NC, HS, AA, and AA inhibitors at the stated time points, and Tukey’s multiple comparison tests was used to determine differences between multiple groups. Data were presented as Means ± SEM. *P* < 0.05 or *P* < 0.01 were considered significant. Results were illustrated in graphs using the GraphPad Prism (GraphPad Software Inc, San Diego, CA).

## Results

### HS upregulated AA level in SCs

**Fig. 1:**
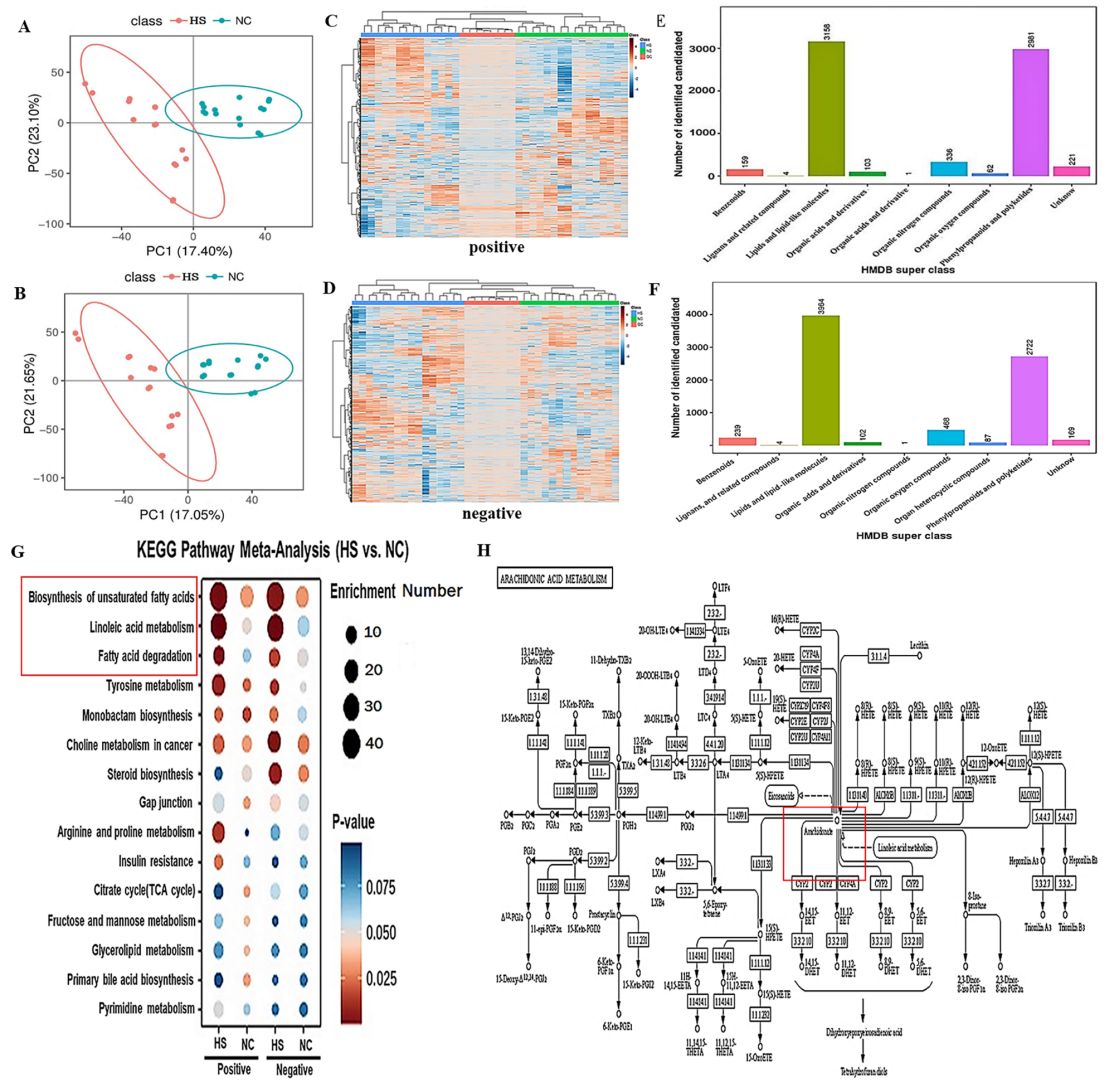
HS upregulated AA level in SCs. (**A-B**) The principal component analysis for NC and HS treatment, orange-red means HS, blue-green means NC; (**C-D**) Metabolic clustering analysis through heat-map for potential homogenized compound selection, blue means HS, green means NC, pink means quality control (QC), each group consists of 8 independent replicates; (**E-F**) Function annotation for differential metabolites in HMDB database; (**G**) Pathway enrichment for differential metabolites in KEGG, each circle means enriched metabolite number, z-score on blue to red scale means *P*-value of per group of metabolite, *P*-value range from 0.025 to 0.075, red present *P*-value ≤ 0.050(up-regulation), white present FC = 0 (without-regulation ) and blue present *P*- value ≥ 0.050 (down-regulation); (**H**) Analysis of metabolic pathway of arachidonic acid in KEGG.

To study the metabolism changes of SCs under heat stress, metabolites was determined by LC-MS/MS analysis. A principal component analysis (PCA) analysis was performed to understand the aggregation and description of the varied treatment samples. The variation in the abundance of metabolites was explained by two principal components (PC). Approximately 17.05-17.4% of the variability in abundance was explained by PC1, while the other components of PC2 explained 21.65-23.10% (Fig. 1A-B). All alterations in levels of important metabolites were clustered using hierarchical clustering analysis with MetaboAnalyst R 3.0 from HS VS.NC samples. Most extracts that were quantified and aligned (with FC ranging from − 4 to + 4), and showed upregulation (Fig. 1C-D, S1-S2 Table). To investigate the functional characteristics of the differentially metabolized metabolites, we examined biological processes and signaling pathways and discovered that multiple metabolites were enriched in the lipids and lipid-like molecules pathway (*N* = 3158 in positive mode and *N* = 3964 in negative mode Fig. 1E-F, ). Notably, we observed these metabolites were mainly enriched in unsaturated fatty acids, linoleic acid metabolism, and fatty acid degradation (Fig. 1G). With a variable importance to projection (VIP) score ≥ 1, FC ≥ 2 or ≤ 1/2and q-value ≤ 0.01, several of these potentially useful biomarkers participate in amino acids, sugars, antibiotic or antioxidant response, hormone and lipid metabolism. Among them, we found that AA was upregulated (about 2.35-fold) in heat stressed SCs, compared to NC, and the significant changes of upstream and downstream metabolites (Fig. 1H; Table 1), suggesting that AA plays an important role during HS, and may exhibit a switch-like response in multiple metabolic pathways during HS.

**Table 1:** The information of metabolite in heat stressed SCs

Name	ID	HS-average	NC-average	FC(HS/NC)	T.test p.value	Bhcorrect Q.value	VIP	CV	Regulated
Arachidonic acid	M379T32	169538.8	72240.1	2.346877	0.000000545	0.000196126	2.511497	2.79E-07	up
Testosterone	M387T35	235711.5	518560.6	0.45455	0.000000432	0.00018375	2.279251	0.083264	down
Prostaglandin	M369T456	9120.337	3423.86	2.663758	0.001460054	0.020502134	2.187894	3.3E-08	up
VB6/Adrenaline	M187T340	13012.78	3402.884	3.824045	0.000112378	0.004307194	2.528222	0.025782	up
D-Glucose	M261T268	2946.518	1234.163	2.387463	0.0016521	0.021946653	2.004409	0.137309	up
Estradiol	M464T39	4696.197	11032.17	0.425682	4.77118E-07	0.000158403	2.430313	0.173357	down
Linoleic acid	M279T88	111404.7	245022.6	0.454671	1.20068E-05	0.001269232	2.357342	0.102976	down
Oligomycin	M821T37	147258.9	325583.5	0.452292	7.67836E-08	5.09843E-05	2.338089	0.059423	down
Glutamine	M274T432	3884.647	1455.857	2.668288	2.83083E-05	0.001988114	2.315496	0.079117	up
VC	M443T560	2731.04	1009.614	2.705033	0.001629079	0.021888794	2.937968	0.224469	up
Leucine	M174T491	1328.259	4305.827	0.30848	0.000632668	0.012685339	2.213439	0.164667	down
Soya fatty acid	M309T88	16225.45	34444.15	0.471065	1.44785E-05	0.001333829	2.253149	0.1023	down
Arginine	M245T296	7442.184	2333.897	3.188737	0.002814327	0.033208152	2.976754	0.245613	up
D-gal	M527T179	7163.57	939.7138	7.623141	0.00175079	0.022835966	2.745514	0.063691	up
Benzoic acid	M199T395	3057.069	1289.126	2.371428	0.001260808	0.018825919	2.170818	0.262047	up
Leukotrienes	M369T456	9120.337	3423.86	2.663758	0.001460054	0.020502134	2.087894	3.3E-08	up
Quercetin	M429T231	528.4834	1691.28	0.312475	6.27204E-08	4.5811E-05	2.966228	0.127685	down
Lysine/Tyrosine	M348T319	3314.474	1647.72	2.011552	0.000268979	0.007622696	2.85249	9E-08	up
Saucernetin	M388T519	1690.254	704.3933	2.399588	0.003287702	0.03659555	2.78215	0.18458	up
Glycerinum/Glycerol	M217T441	766.2402	3739.17	0.204923	0.00526372	0.043267748	2.408983	0.095788	down

### HS induced autophagy in SCs via AA

**Fig. 2:**
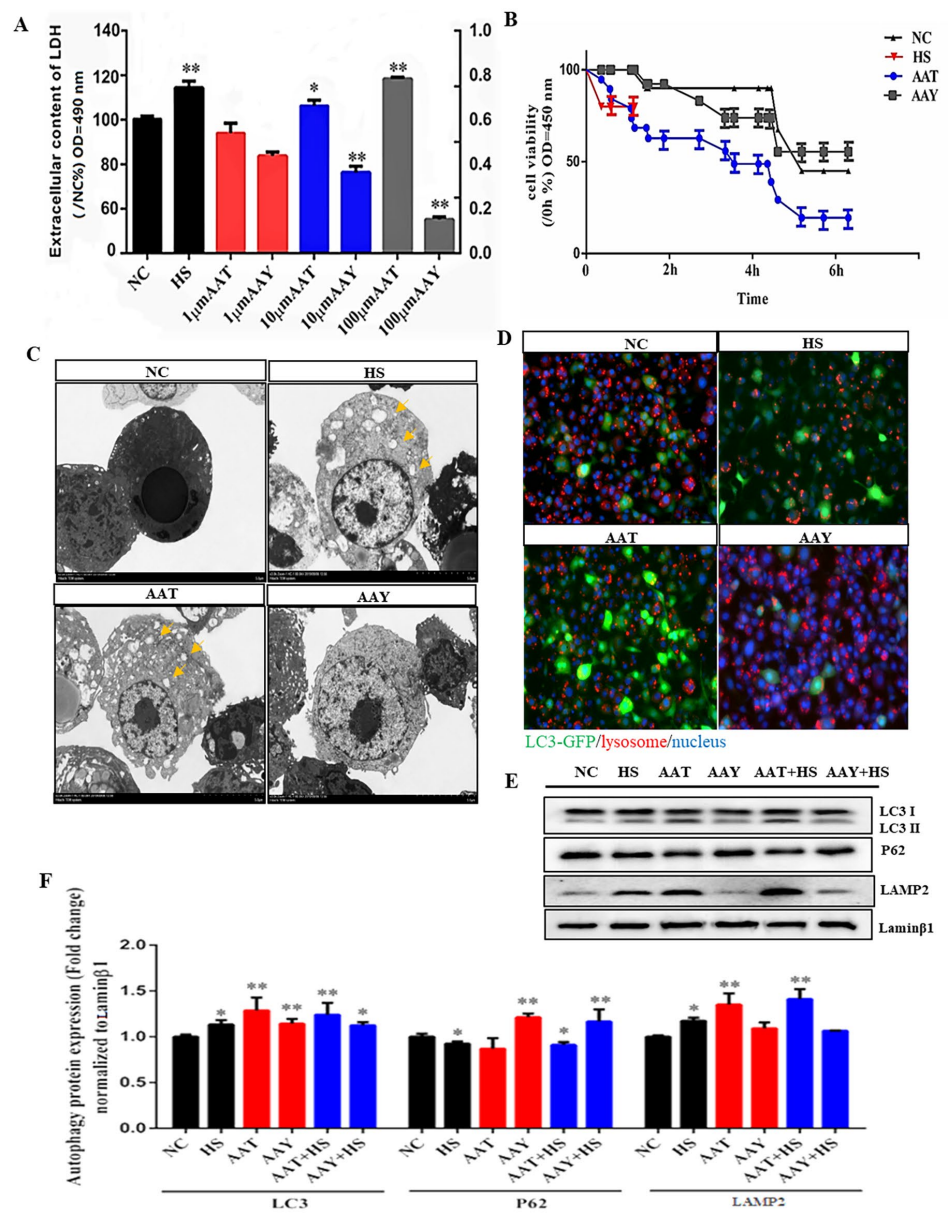
HS induced autophagy in SCs via AA. (**A**) Toxicity determination by LDH of SCs after addition/inhibition of AA, data are expressed as mean ± SEM, * *P* < 0.05, **P < 0.01 means significant difference between indicated concentration of AA addition vs. without supplement of AA; (**B**) SCs viability detection after addition/inhibition of AA, NC: Normal group; HS: Heat stressed group; AAT: Arachidonic acid group; AAY: Arachidonic acid metabolism inhibitor group. data are expressed as mean ± SEM, * *P* < 0.05, **P < 0.01 showed that significant difference between indicated treatment time of HS/AAT/AAY vs. NC; Values are mean ± standard error (technical replicates *n* = 8, 3 independent replicates for per trial), * or ** indicates that there are significant differences between each treatment group and normal group, the same as below; (**C**) Effects of AA on autophagy measured by TEM; (**D**) Immunofluorescence detected autophagic lysosome formation and distribution in SCs, red means lysosome, green means autophagosome, blue means nucleus, the same as below; (**E-F**) Effects of AA on expression of autophagy related proteins, measured by LC3, p62 and LAMP2, normalized by Lamin β1

To determine the effect of AA on the number of SCs under HS, we investigated cell viability and cytotoxicity under the treatment of AA. Cells were treated with varied concentrations of AA (AAT, 10∼100 µM) and its inhibitor (AAY, 10∼100 µM). In parallel with the increase of AAT concentration, relative LDH release peaked at 100 µM of AA (*P* < 0.05), indicating that 100 µM of AAT can mimic the effect of HS on cell viability and cytotoxicity. In contrast, the LDH content in the 100 µM of AAY group was lower than that of the NC group (*P* < 0.01, Fig. 2A). Meanwhile, with the increase of the treatment time (0 to 6 h) for the 100 µM AAT and HS (0 to 1 h), cell viability declined compared to the control, whereas the cell viability increased in the AAY (*P* < 0.01, Fig. 2B). Therefore, the treatment of 100 µM AAT or AAY for 6 h was chosen in a subsequent experiment. In TEM images, the autophagosomes were observed under the exposure of HS and AAT. We found a vacuole-like structure of bilayer containing cytoplasmic components in those two groups (Fig. 2C). In line with this, the immunofluorescence analyses showed that compared to NC group, positive-GFP LC3 was observed to site in SCs under HS group. Also, intensity of autophagy-lysosome staining was stronger in AAT group, while the AAY inhibited the formation of autophagolysosome (Fig. 2D). Western blot data showed that AAT increased the autolysosome presence in initially HS with a consequence of a transformation in LC3-I to LC3-II protein ratio and the ratio increases to 1.25-fold (*P* < 0.05), an increase in LAMP2 protein expression in 1.4-fold (*P* < 0.05) and a significantly decrease in the expression of P62 in 0.3-fold (*P* < 0.05, Fig. 2E-F), indicating that both of AA and HS can induce the formation of autophagolysosome by activating autophagy.

### HS activated OS and induced ROS generation via AA

**Fig. 3:**
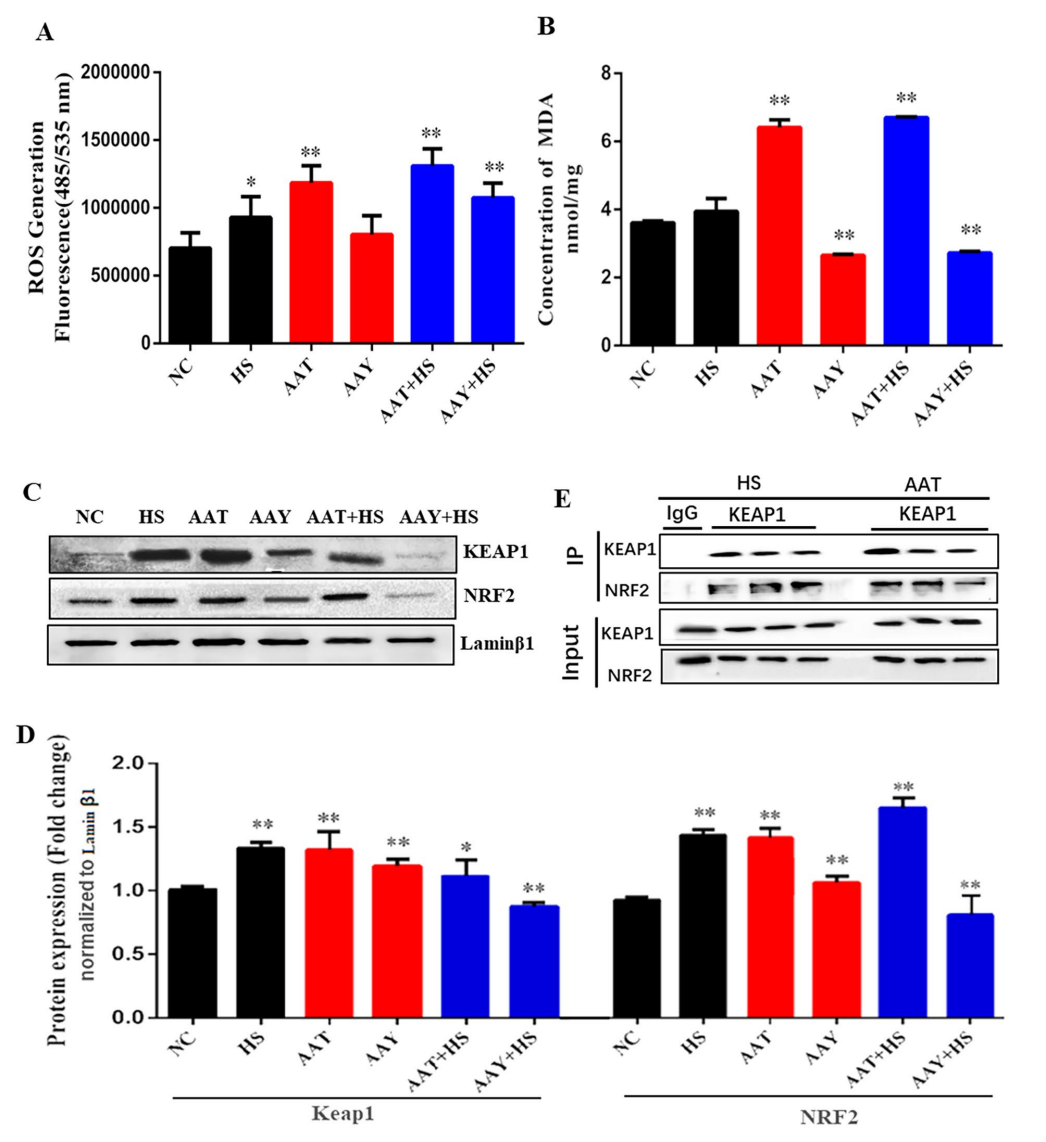
HS activated OS and induced ROS generation via AA. (**A**) Changes of ROS concentration after addition/inhibition of AA; (**B**) Changes of MDA concentration after addition/inhibition of AA; (**C-D**) Changes of OS related genes KEAP1 and NRF2 expressions were detected by western blot; (**E**) Interaction between KEAP1 and NRF2 is combined after HS and AA treatment

To determine the mechanism of autophagy activated in response to changes in AA during HS. Our first step was to examine OS parameters, as excessive release of unsaturated fatty acids is known to trigger lipid peroxidation, potentially initiating autophagy [28, 29]. We observed that HS and AAT increased the concentrations of MDA and ROS in the range of 0.5∼2-fold, exhibiting synergistic effects compared to NC *(P* < 0.05, Fig. 3A-B). Furthermore, an increased protein expression of Kelch-like ECH-associated protein-1 (KEAP1) and Nuclear factor erythroid 2-related factor 2 (NRF2) were observed (approx.0.5-fold increase, *P* < 0.05, Fig. 3C-D). Consistently, the co-immunoprecipitation of KEAP1 revealed that AA activates the interaction between KEAP1 and NRF2 (Fig. 3E), suggesting that AA could activate OS during HS.

### HS injured mitochondrial structure via AA

**Fig. 4:**
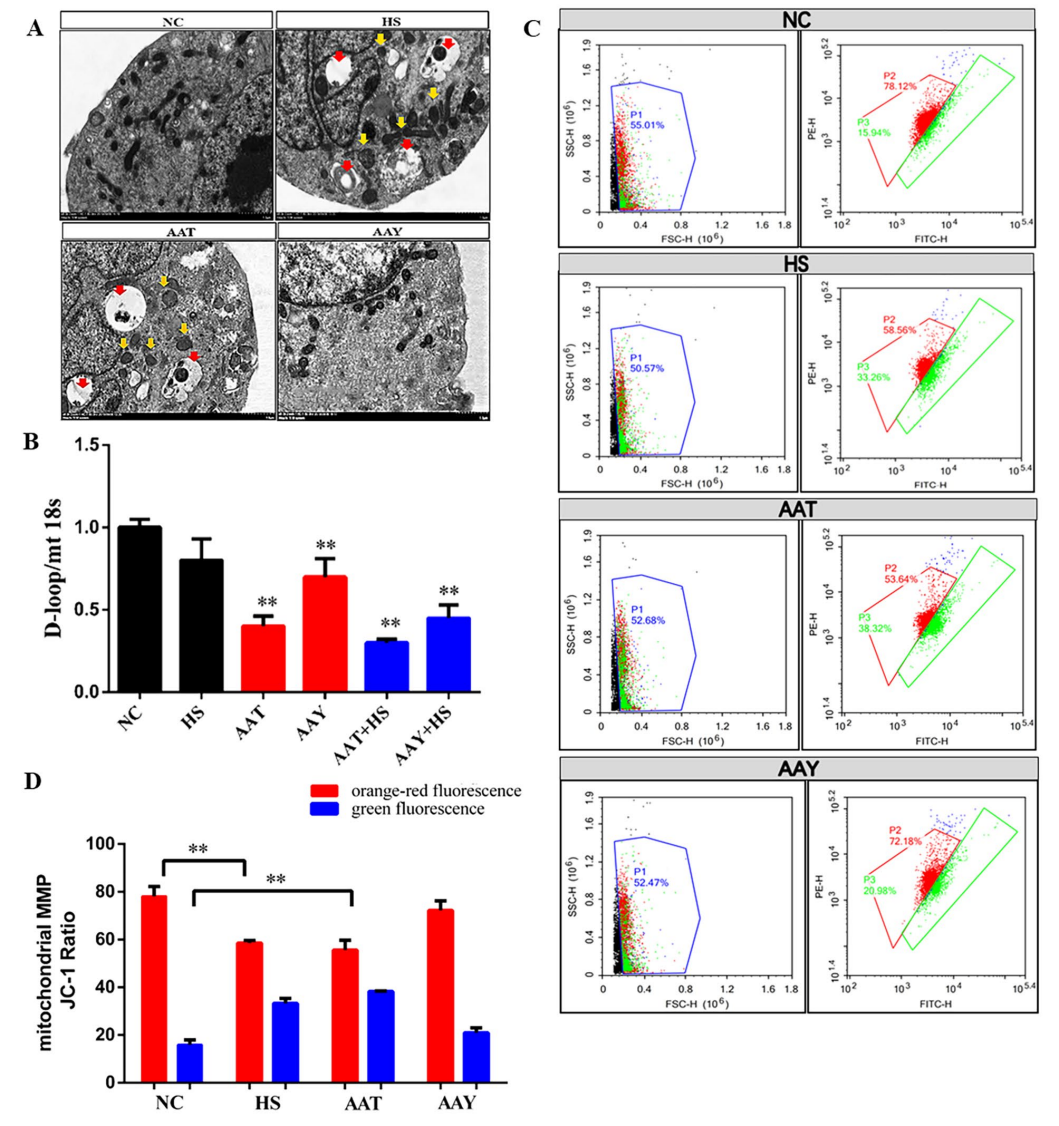
HS injured mitochondrial structure via AA. (**A**) Effects of AA on autophagosome distribution and mitochondrial vitality in SCs (red arrow means autophagosome, yellow arrow means mitochondrial); (**B**) Expression changes of D-loop in mitochondria after AA supplementation/inhibition; (**C-D**) Changes in MMP after addition/inhibition of AA, Cells staining with orange-red fluorescence indicate that the MMP of the SCs is normal

The next step was to determine mitochondrial structure and function as ROS is associated with impairments in the oxygen transport and absorption functions of mitochondria. We found that the mitochondria of control group were normal, while swollen mitochondria were observed in AA and HS treatment group (yellow arrow). Post exposures of NC or AAY to SCs, mitochondria were normal with no apparent autophagic vesicles in their cytoplasm, whereas AAT and HS induced autophagic vesicles (red arrow, Fig. 4A). HS (approx.0.3-fold) and AA (approx.1.2-fold) exposed to cells resulted in a significant decrease in mitochondrial D-loop (a maker could indicate the mitochondrial abundance) content; however, the addition of AAY partially reversed this phenomenon (Fig. 4B). JC-1 aggregates in the mitochondria emit orange-red fluorescence. Normal SCs present high mitochondrial polarization. After HS and AAT-induced dysfunction of mitochondria, we observe a loss in MMP (ΔΨm) with an increased green fluorescence, the ratio was 34.82 ± 2.45 VS.17.58 ± 2.78, compared to NC (*P* < 0.05, Fig. 4C-D).

### HS dysfunction the ETC via AA

**Fig. 5:**
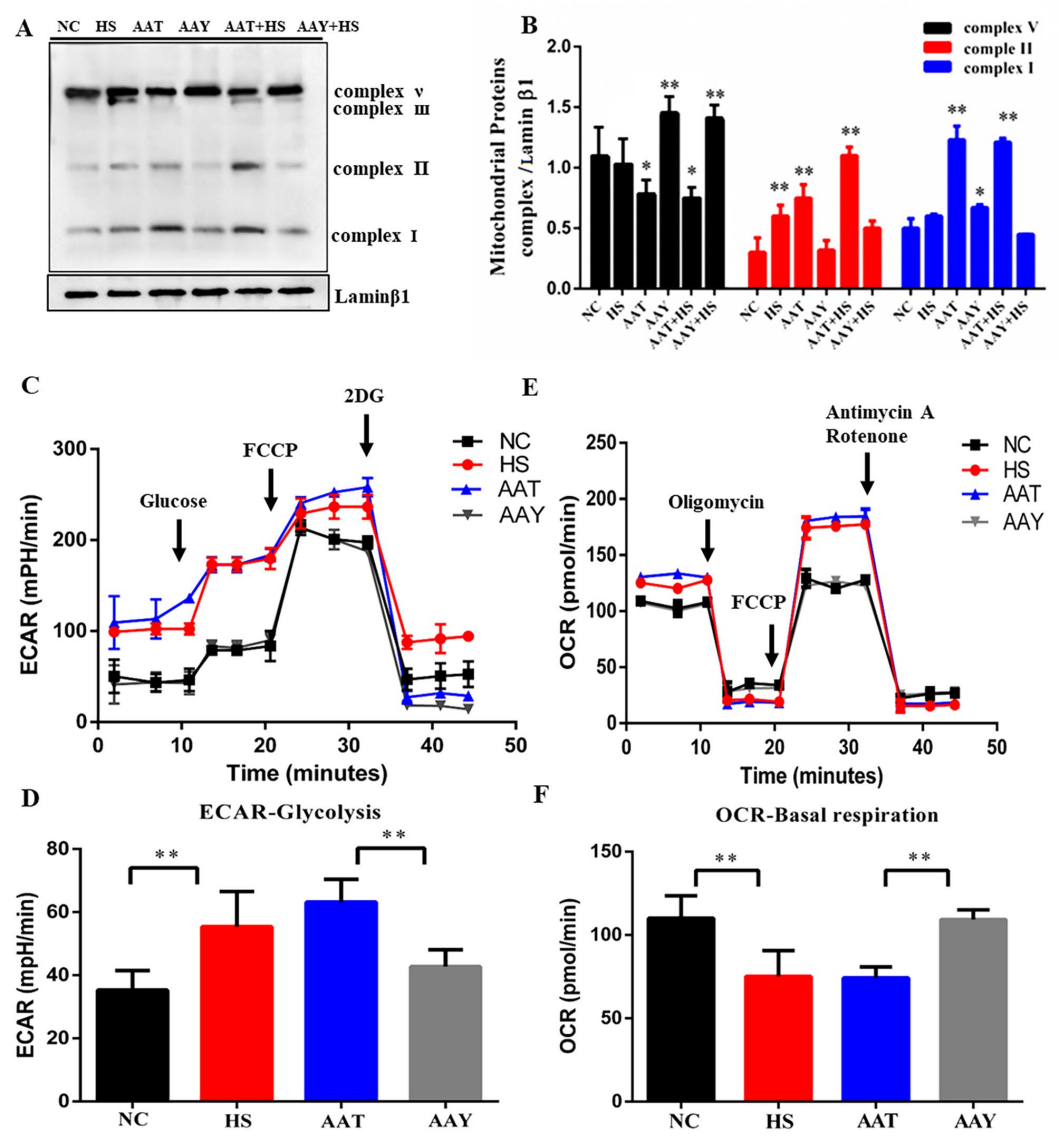
HS dysfunction the ETC via AA. (**A-B**) Changes of mitochondrial respiratory chain protein expression and quantification after AA supplementation/inhibition; (**C-D**) The ECAR was detected to indicate glycolysis flux; (**E-F**) The OCR was detected to indicate basal respiration

The loss of MMP is associated with the metabolic shift from OXPHOS to glycolysis in SCs, we then aimed to examine the changes in expression of the mitochondrial ETC protein-Complex I-V. In comparison with the NC group, HS increased the expression of Complex I, II and V (approx.0.1∼0.3 fold, *P* < 0.05). Similarly, those proteins aggravated in AAT treatments (approx.0.5∼1.0 fold, *P* < 0.05, Fig. 5A-B). The Seahorse XF Cell was used to measure ECAR and OCR, serving as indicators of glycolysis flux, OXPHOS and basal respiration levels, respectively [30]. The steady state glycolysis flux (lower panels) increased in both HS and AAT, indicating that HS and AAT promote glycolysis flux and glycolytic capacity. However, HS and AAT significantly inhibited glucose uptake while promoting lactate production (Fig. 5C-D). Seahorse analysis revealed a decreased oxygen consumption rate (OCR) in HS and AAT. On the contrary, NC and AAY groups showed that mitochondria had normal levels of state I- III ECAR and state Ӏ-III OCR (Fig. 5E-F). As expected, AAY completely reversed the decreased glucose uptake and increased of lactate production in ECAR. Together, the results suggest that upregulation of AA in HS conditions promotes glycolysis, while decreasing mitochondrial OXPHOS, which would promote ROS generation.

### AA induced autophagy was partly alleviated by NAC

**Fig. 6:**
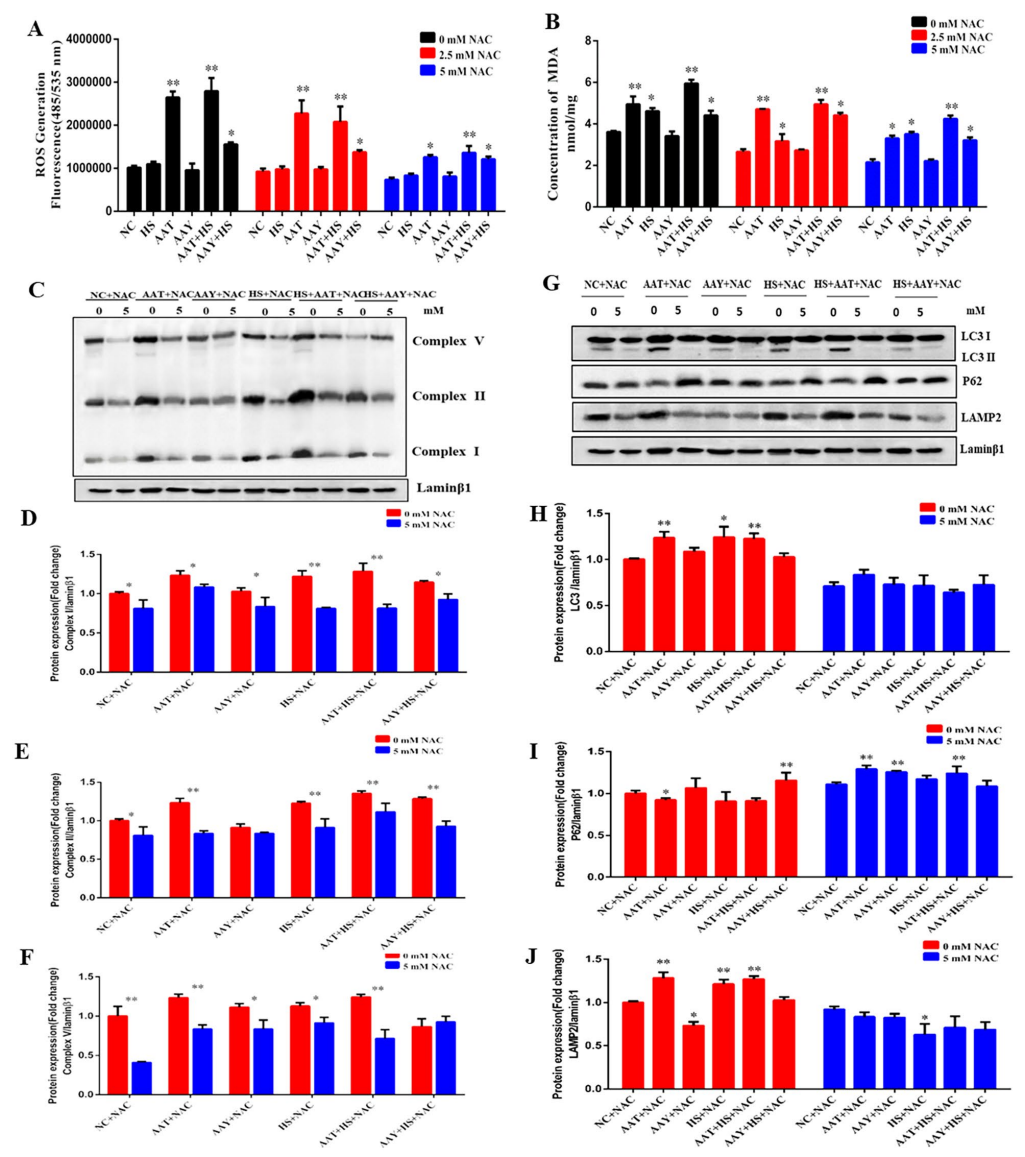
AA induced autophagy was partly alleviated by NAC. (**A**) Role of NAC in AA-induced ROS production; (**B**) Role of NAC in MDA production induced by AA; (**C-F**) Effect of NAC on mitochondrial respiratory chain protein expression after AA supplementation or HS exposure; (**G-J**) Effect of NAC on autophagy protein (LC3\P62\LAMP2) expressions after AA supplementation or HS exposure

To clarify HS-induced autophagy in SCs in response to ROS induced by AA, three concentrations of NAC (0 mM, 2.5 mM and 5 mM), as an ROS inhibitor, were added. Comparative analysis revealed a significant reduction in ROS and MDA when using 5 mM NAC compared to with 0 mM and 2.5 mM NAC (*P* < 0.05, Fig. 6A and B). Additionally, 5 mM of NAC inhibited the activity of complexes Ӏ, II and V, leading to a decrease in their protein expressions ranging from 0.2-fold to 0.8-fold (*P* < 0.05, Fig. 6C-F). Moreover, the protein expression of LC3II/Ӏ and LAMP2 significantly decreased in both HS and AAT groups after the addition of NAC, while P62 protein expression increased (*P* < 0.05 Fig. 6G-J).

### AA induced autophagy was partly reversed by Rotenone

**Fig. 7:**
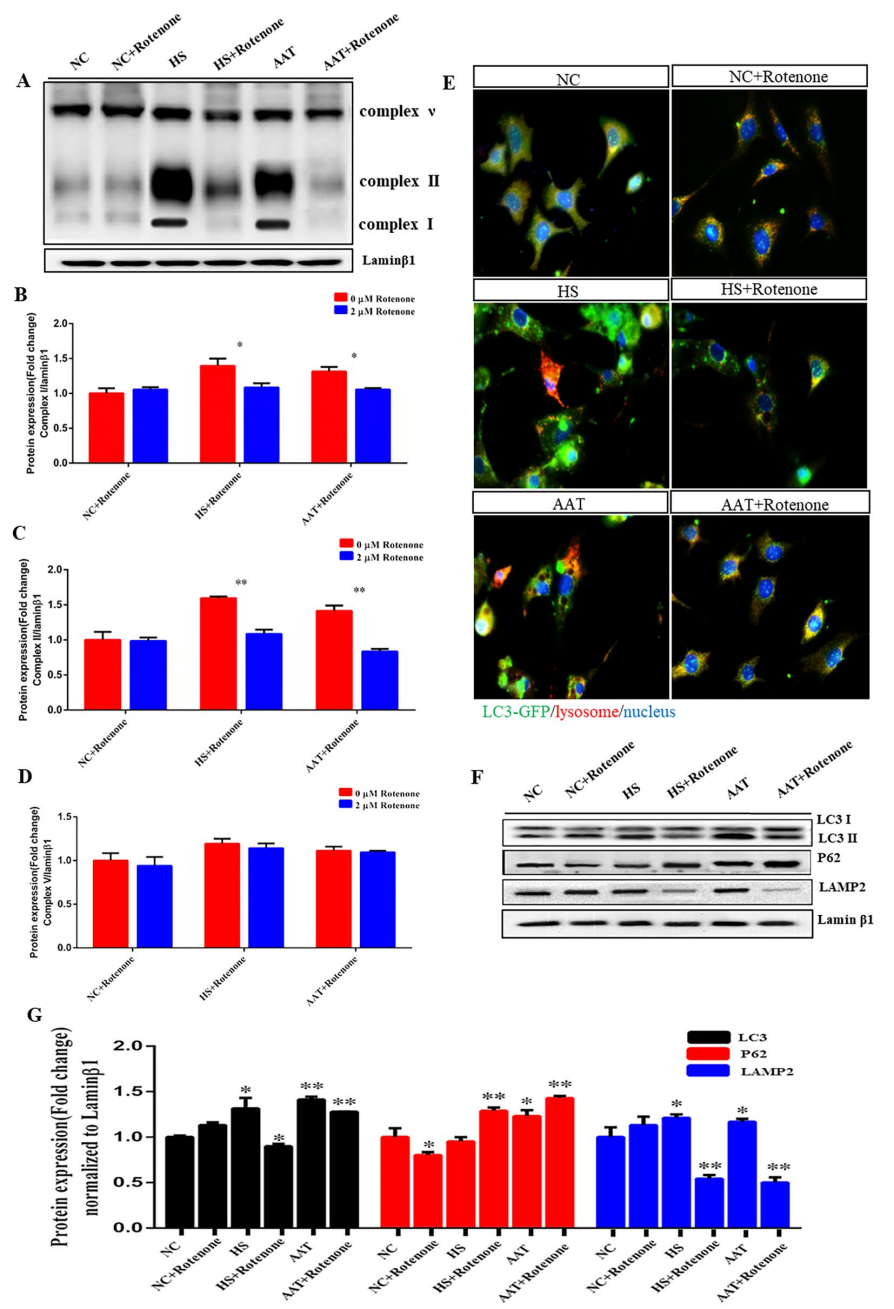
AA induced autophagy was partly reversed by Rotenone. (**A-D**) Rotenone changes the mitochondrial ETC protein expression upon AA stimulation; (**E**) Role of Rotenone in AA-induced changes on localization and abundance of autophagic lysosomes; (**F-G**) Role of Rotenone in AA-induced expression of autophagic protein LC3\P62\LAMP2

To illustrate the role of mitochondrial function in regulating autophagy, Rotenone, a mitochondrial ETC Complex I inhibitor was used in the AA treatment group. The protein expression of Complex I and II significantly decreased both in HS and AA groups compared to NC, ranging from o.3-fold to 0.8-fold, while the addition of AAY could partially reverse the decline (Fig. 7A-B). Rotenone blocked the increase in proteins expression of LC3 and LAMP2 induced by AA and HS, but lead to a decrease in P62 (Fig. 7F-G, *P* < 0.05). The immunofluorescence data showed that LC3-II levels were slightly reduced in SCs in NC + Rotenone conditions but were dramatically reduced when cells were treated with AAT + Rotenone and HS + Rotenone conditions (Fig. 7E). These results indicate that autophagy induced by AA under HS is regulated by mitochondrial ETC, which are link to OS activation and ROS generation. Inhibition of these processes may reverse the phenomenon of autophagy.

## Discussion

In this study, we observed that HS induced autophagy in the SCs. We initially demonstrated that HS enhanced the content of AA, a fatty acid derived from cell membrane phospholipids. Both HS and AA elevated ROS and MDA concentrations in cells, upregulated protein expression in KEAP1 and NRF2, and triggered OS. Moreover, the impairment of mitochondrial ultrastructure and function by HS or AA led to upregulated glycolysis, decreased oxygen consumption rate, and reduced the mitochondrial D-loop DNA as well as the increase of proteins expression of Complex I, II, V. In mitochondria, the electrical leak, in conjunction with oxygen, stimulates ROS production, subsequently inducing autophagy, as evidenced by alterations in LC3, P62, and LAMP2 abundance, suggesting a crucial role of AA in cellular responses to HS. Notably, supplementation with NAC and Rotenone reversed the AA-induced autophagy. The results inferred that an intriguing link between AA and SCs viability, regulated by mitochondrial ETC function. The study provides valuable insight into the development of HS-related male reproductive diseases.

SCs play a crucial role in supplying germ cells with polyunsaturated fatty acids (PUFAs) in the testis. A comprehensive understanding of the metabolism alteration of SCs disrupted by HS could provide important insights into preventing the harmful effects of lipid peroxide products on spermatogenesis. In our preliminary experiment, we observed a significant upregulation of AA in heat-treated SCs (Fig. 1). Derivatives of AA such as leukotrienes and prostaglandin, were also detected, which is consistent with the fact that heat shock (42–45 °C) increased AA release from various cell types, including human, rat, murine, and hamster cells [31], inferring that AA could be a potential biomarker for assessing SCs’ response to HS. To mimic the effect of HS, we evaluated the optimal concentration of the AA which could replicate HS effects, and found that treating cells with 100 µM AA for 6 h significantly impacted cell proliferation as evidenced by intracellular LDH and cell viability detection (Fig. 2A-B). Although AA typically acts as a nutrient and plays a vital role in cellular development [32], for the first time our data demonstrated that abundant AA significantly inhibited cell growth, and induced autophagy vesicle formation (Fig. 2C). To prove with this, we observed an increase in the abundance and membrane presence of lysosome and LC3-GFP, which could be reversed by AAY (Fig. 2D). Additionally, AA also increased the expression of autophagy-related proteins LC3 and LAMP2, while decreasing the expression of P62 (Fig. 2E-F). Consistent with our findings, traumatic brain injury (TBI) -induced lysosomal membrane permeability was linked to PLA2G4A/cPLA2 (phospholipase A2, the gene is responsible for regulating arachidonic acid) lipid profiles based on LC-MS/MS analysis of the lysosomal membrane lipid profiles in TBI and sham animals [33]. Komiya et al., found that Palmitate, a saturated fatty acid, induced autophagy in neurons, muscles, and liver cells by strongly increasing the conversion of LC3I to LC3II [34]. Xu et al., reported that an increase in autophagosomes, lysosomes, and autophagy flux may be observed in raw264.7 cells in various concentrations of linoleic acid or docosahexaenic acid [35]. This implied that both saturated and unsaturated fatty acids can trigger autophagy, and their roles in cell functions were affected by cellular conditions.

It is reported that HS induces lipid peroxidation in avian liver and 3T3-L1 preadipocytes, concurrently promoting lipid accumulation [36, 37]. Lipids peroxidation played a role in HS tolerance as well as decreased activity of antioxidant enzymes in both plants and animals [38, 39]. The PUFAs could undergo free radical chain reactions during stress in vitro, leading to the accumulation of ROS [40]. In our study, we found that both HS and AA increased concentrations of MDA and ROS with synergistic effects when two were combined (Fig. 3A-B). A consistent increase in KEAP1 and NRF2 protein expression was observed after exposure to the two groups (Fig. 3C-D), indicating that the KEAP1-NRF2 system plays a major role in OS response. Besides being a receptor for NRF2, KEAP1 also serves as an adapter of NRF2. We found that both HS and AA promote interaction between KEAP1 and NRF2 (Fig. 3E), suggesting that AA might induce the accumulation of ROS when exposed to HS. This may be attributed to the unsaturated bond in the sn-2 position in AA. HS alters the configuration of sn-2 phospholipid membrane, resulting in AA to modify membrane fluidity, furthermore leading to fatty acid desaturation and increased susceptibility to lipid peroxidation, ultimately activating OS [41, 42].

ROS are generated through three mechanisms: mitochondria, endoplasmic reticulum and cytoplasm. We then assessed functional changes in mitochondrial biogenesis. The result indicated an increase of mitochondrial glycolysis process and ETC proteins (e.g., Complex I, П, V) during HS. These effects were intensified by AAT (Fig. 5). However, the MMP and D-loop exhibited opposing changes in those two groups (Fig. 4). In line with this, Yan et al., reported that HS enhanced the breakdown of unsaturated fatty acids and accelerated glycolysis and tricarboxylic acid cycle in P. ostreatus [43]. Tian et al., demonstrated that COX-mediated AA derivatives Prostaglandin 2α promote autophagy, improve mitochondrial accumulation and ATP production, and increase the transcription of genes involved in oxidative metabolism and mitochondrial ETC in fish hepatocytes [44]. This is because that fatty acid can induce mitochondrial dysfunction by accelerating glycolysis process, and causing a portion of oxygen to be converted to superoxide. As a consequence, autophagy can be activated via pro-oxidant compounds (H_2_O_2_) and decreased oxygen pressure.

Next, we pretreated cells with 5 mM N-acetylcysteine and 2 µM Rotenone following exposure to AA and HS. Our findings clearly demonstrated that NAC reversed the autophagy induced by AA in SCs (Fig. 6). The immunofluorescence data revealed that Rotenone resulted in a partially reduction in lysosomes and LC3-positive fluorescence distribution, as well as proteins LC3 and LAMP2 expression (Fig. 7E-G), indicating that AA might play a crucial role in triggering autophagy under HS by disrupting mitochondrial ETC. The likely reason is that ROS generated as by-products of oxygen metabolism in the mitochondrial Complex I, induce autophagy activation when nutrients such as glucose, amino acids, or serum are depleted under stress [45, 46]. Inceoglu et al., reported that the release of epoxy fatty acids could stabilize the mitochondrial-ROS-endoplasmic reticulum stress (ERS) axis, preventing mitochondrial dysfunction, reducing Ca^2+^ transportation, and averting unfolding protein response [47]. This suggests that different cell types exhibit the distinct metabolic responses to HS, while the raising question is whether Ca^2+^ is involved in AA-induced autophagy because Ca^2+^ could regulate the electron transfer, membrane potential and metabolism of mitochondrial [48]. Furthermore, AA directly modulates inflammatory responses by modulating the activity of TLR4/MyD88 in H9C2 and RAW264.7 cell lines [49]. Autophagy reduces inflammatory responses in macrophages via controlling the TLR4/MyD88 pathway [50]. Therefore, we will investigate the effect of Ca^2+^ transfer or inflammation response on autophagy under AA treatment in the future study.

In summary, our current work has revealed a molecular pathway implicated in heat stress-induced damage in SCs with a unique focus on of AA’s function in triggering autophagy in SCs under heat. However, there are some limitations in the present study: 1) The selection of animal models for toxicity assessment is typically based on convenience rather than validity for human use. The current study is conducted only in vitro, as it can be challenging to discern normal physiology role from pathological role in vivo model. 2) This metabolome analysis is conducted using non-targeted research. Unlike targeted metabolome analysis, which focuses on some known metabolites to discover new biomarkers, non-targeted metabolome analysis can identify a larger number of metabolites. However, the detection may have weak specificity, resulting in fewer biomarkers being identified as candidate organisms.

## Conclusion

**Fig. 8:**
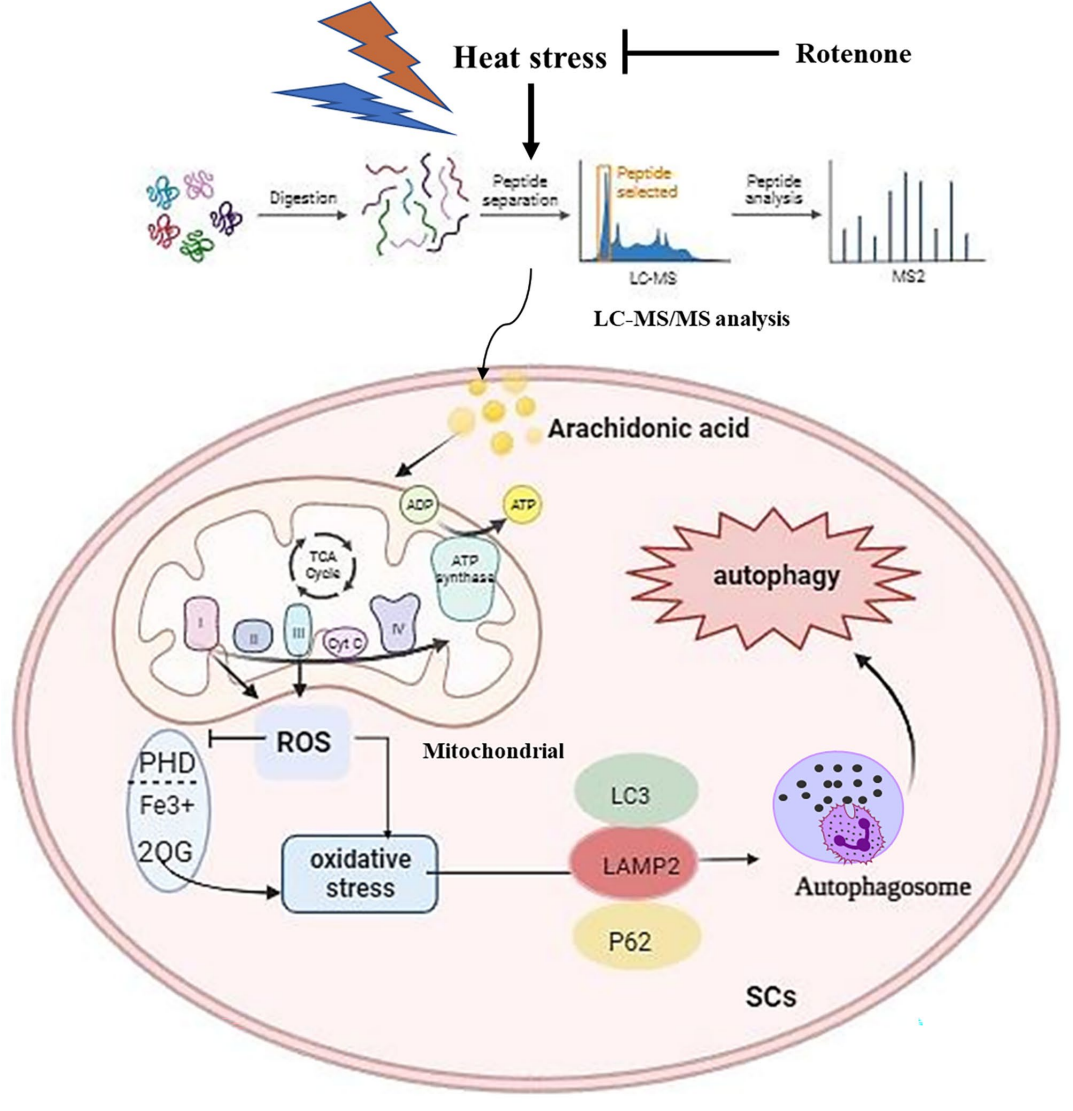
Potential mechanism of AA induced autophagy during HS condition. SCs are influenced by the level of free fatty acids AA through HS exposure, which results in an increased production of lipid peroxides and the generation of ROS, causing OS and then damages mitochondrial structure impaired mitochondrial ETC, even induce mitochondrial stress, which in turn promotes ROS accumulation and increases the likelihood of cell death. Finally, upregulated the proteins LC3 and LAMP2 expression, activate autophagy. However, it may be possible to reverse via inhibiting AA production or adding Rotenone

These findings represent novel insight into the physiological mechanism between autophagy and mitochondria ETC axis in response to HS-induced AA release. This process is suspected to mediate the activation of mitochondrial Complex I protein in response to AA-induced OS. Clearly, during AA stimulation, the accumulation of ROS or toxic aggregates results in a decrease in ETC protein and enzyme activities in mitochondria, which disturb the energy balance between mitochondrial fatty acid oxidation and glucose oxidation (including reduced mitochondrial uncoupling). Consequently, this can reduce the permeability and potential disruptions of mitochondrial membranes, impair mitochondrial homeostasis, which in turn accelerate ROS generation and ultimately trigger autophagy. These results inferred that the release of AA is a switch-like response. Furthermore, the study suggests that Rotenone could hold promising therapeutics for preventing autophagy caused by AA during HS (Fig. 8).

### Electronic supplementary material

Below is the link to the electronic supplementary material.

Supplementary Material 1

Supplementary Material 2

Supplementary Material 3

Supplementary Material 4

## Data Availability

Not applicable.
