# Supplementary material for: Heat stress upregulates arachidonic acid to trigger autophagy in sertoli cells via dysfunctional mitochondrial respiratory chain function
Source: J Transl Med. 2024 May 26;22:501. doi: 10.1186/s12967-024-05182-y (PMC11129461; doi:10.1186/s12967-024-05182-y)
Supplement: Supplementary file 3 — Supplementary Material 3 [file 12967_2024_5182_MOESM3_ESM.pptx]

## Slide 1
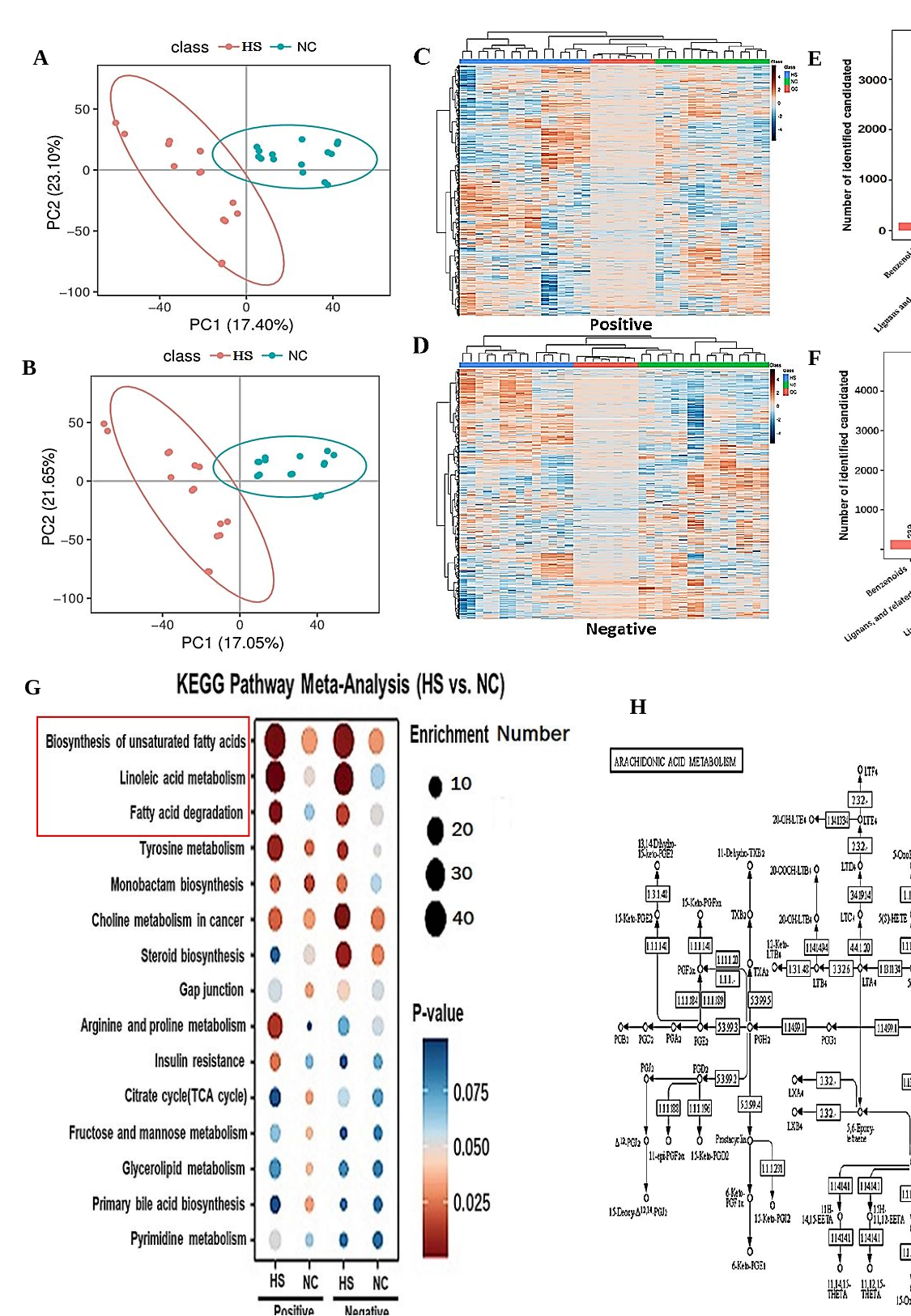

E
F
A
G
H
B

## Slide 2
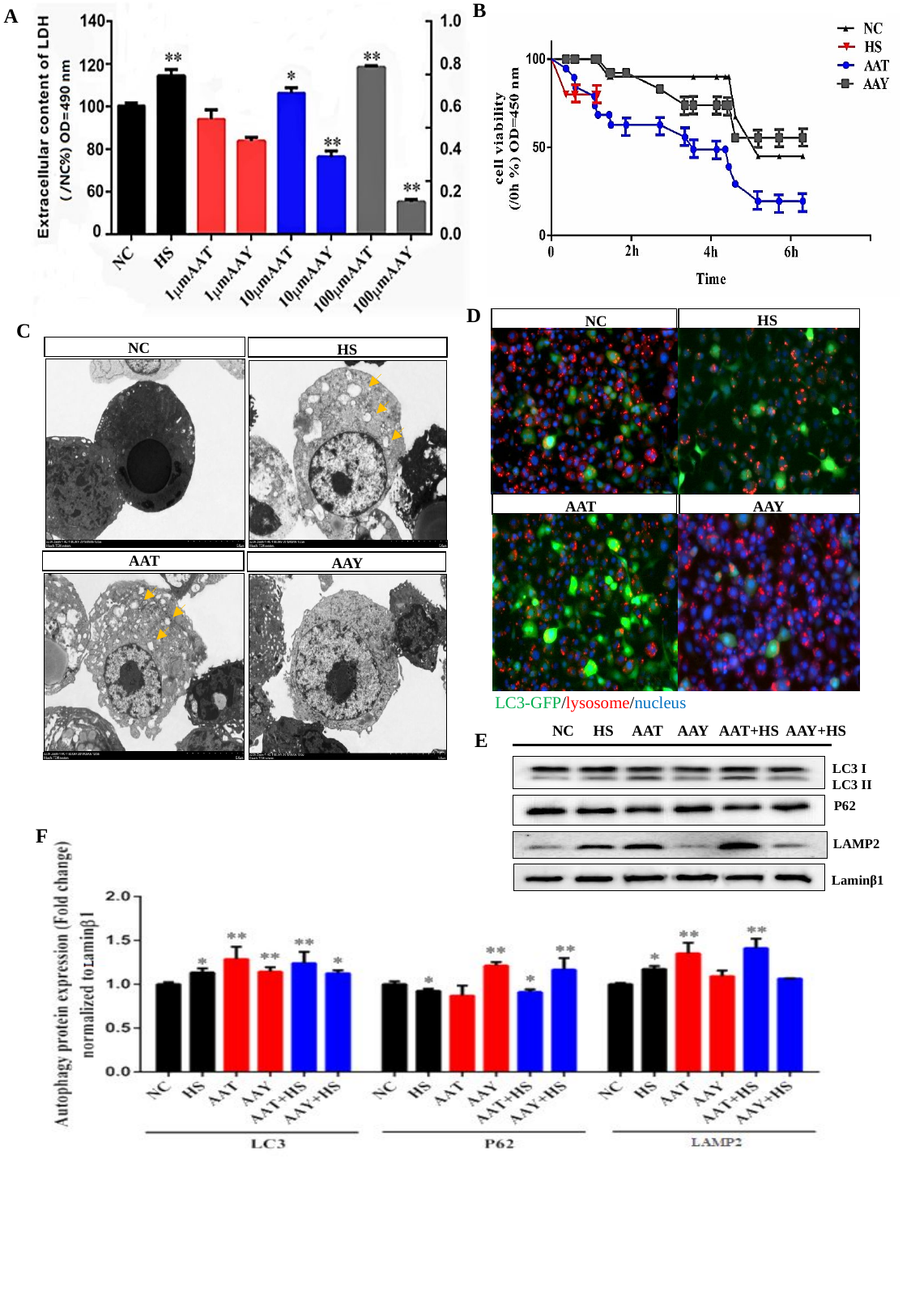

B
A
D
HS
NC
AAT
AAY
LC3-GFP/lysosome/nucleus
C
NC
HS
AAT
AAY
 NC HS AAT AAY AAT+HS AAY+HS
LC3 I
LC3 II
P62
LAMP2
Laminβ1
E
F
AAT
AAY

## Slide 3
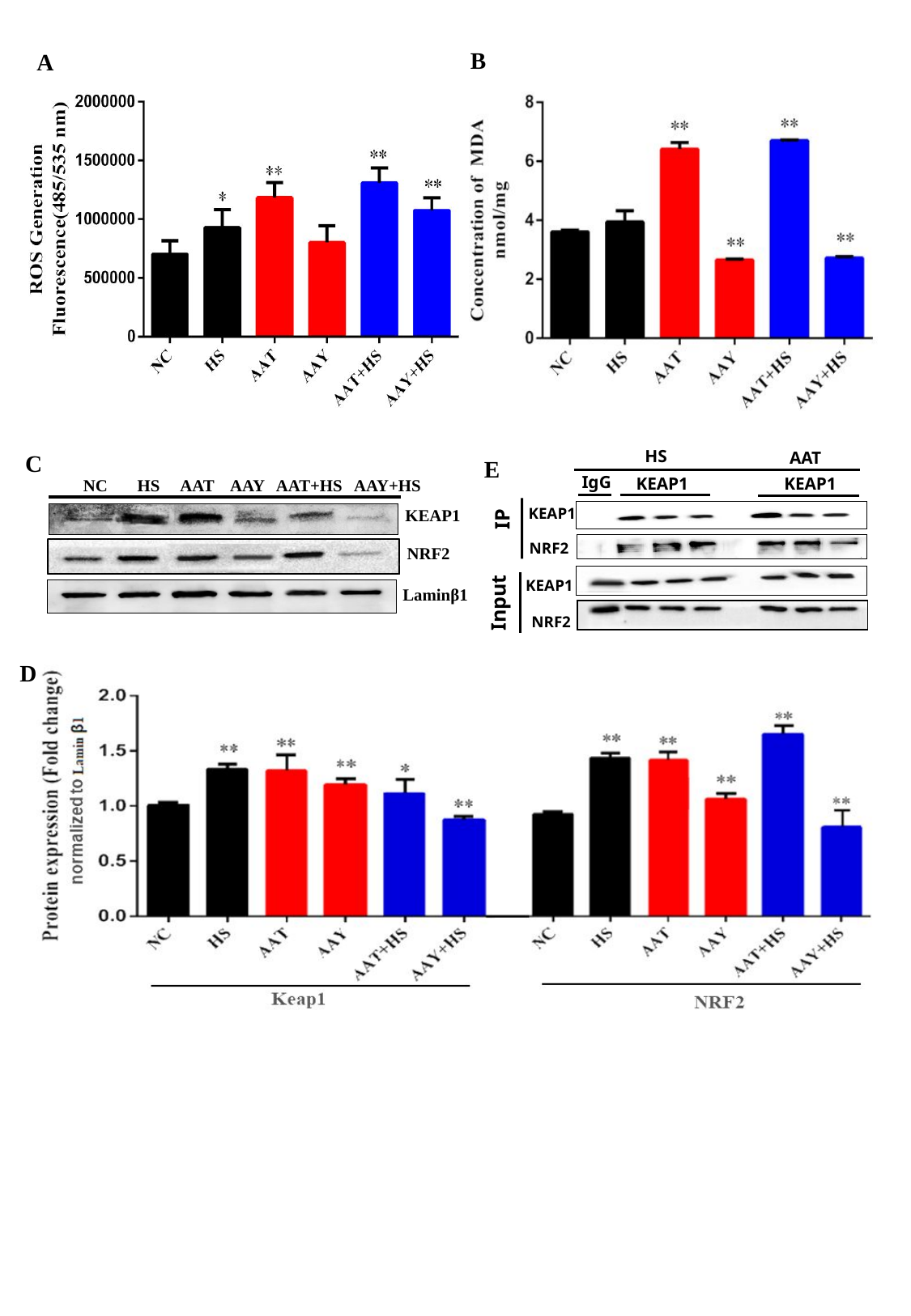

B
A
HS
AAT
IgG
KEAP1
KEAP1
KEAP1
IP
NRF2
KEAP1
Input
NRF2
E
C
 NC HS AAT AAY AAT+HS AAY+HS
KEAP1
Laminβ1
NRF2
D

## Slide 4
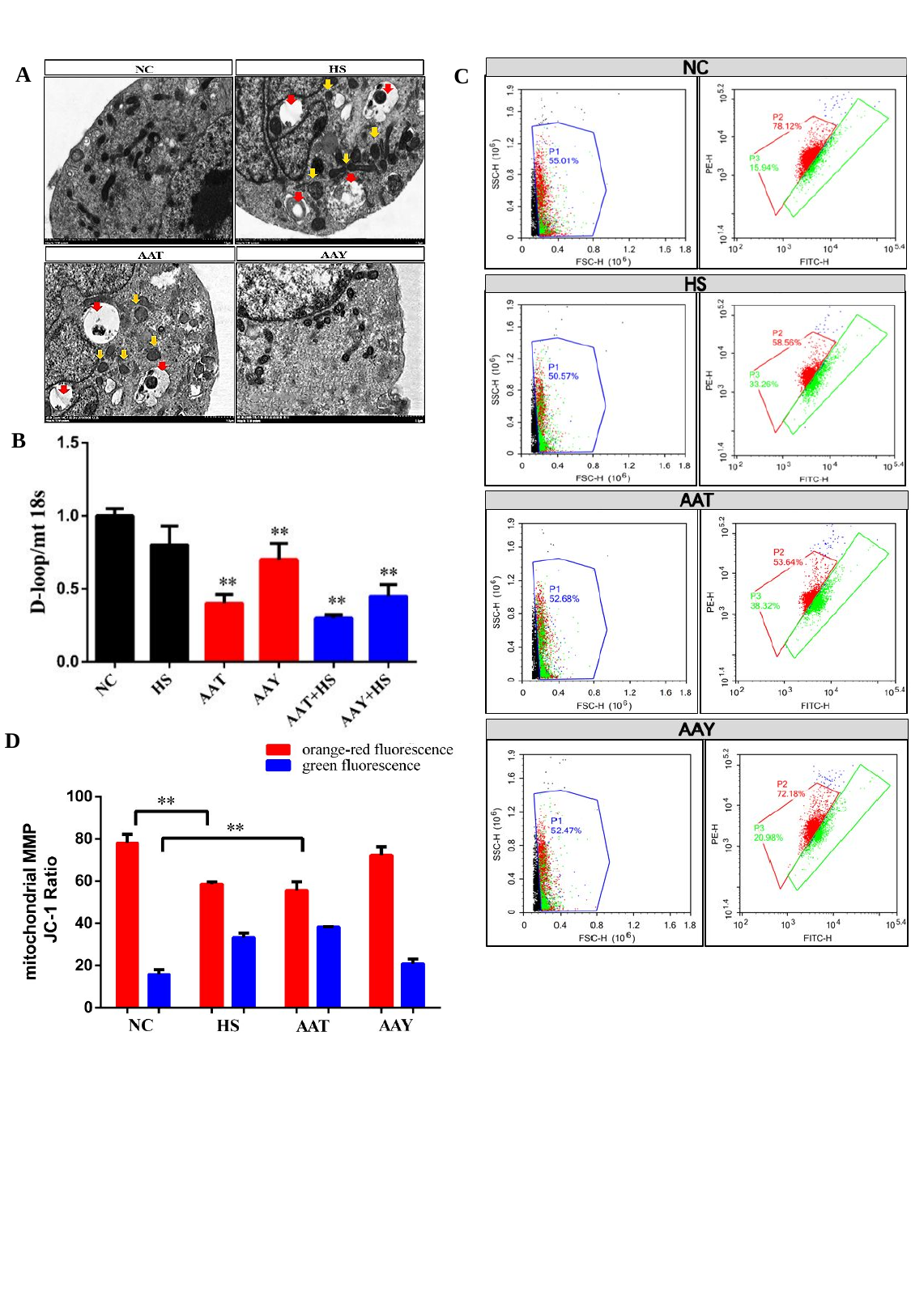

C
A
B
D

## Slide 5
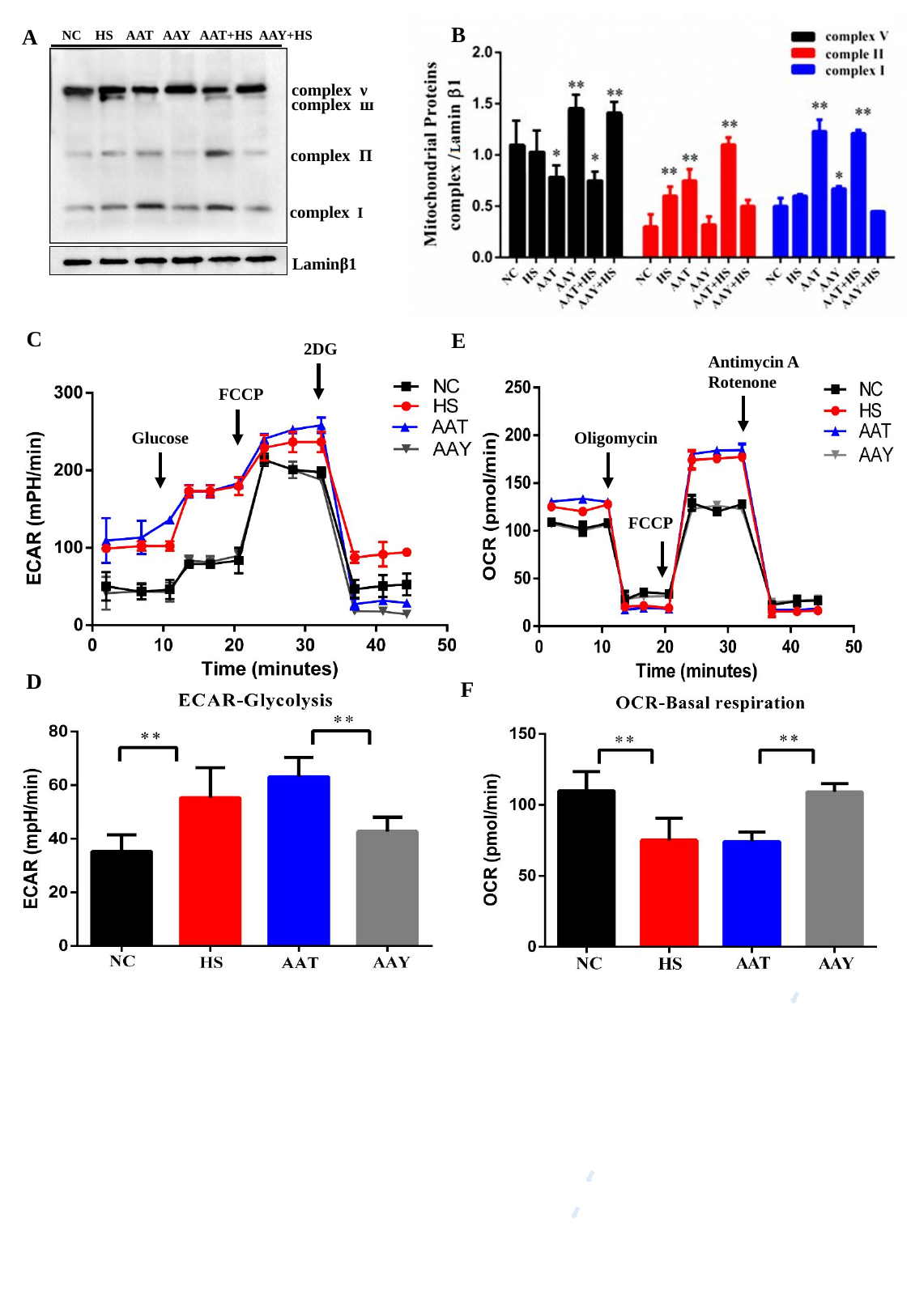

A
 NC HS AAT AAY AAT+HS AAY+HS
complex ν
complex ш
complex П
complex Ӏ
Laminβ1
C
2DG
FCCP
Glucose
E
Antimycin A
Rotenone
Oligomycin
FCCP
D
F
B

## Slide 6
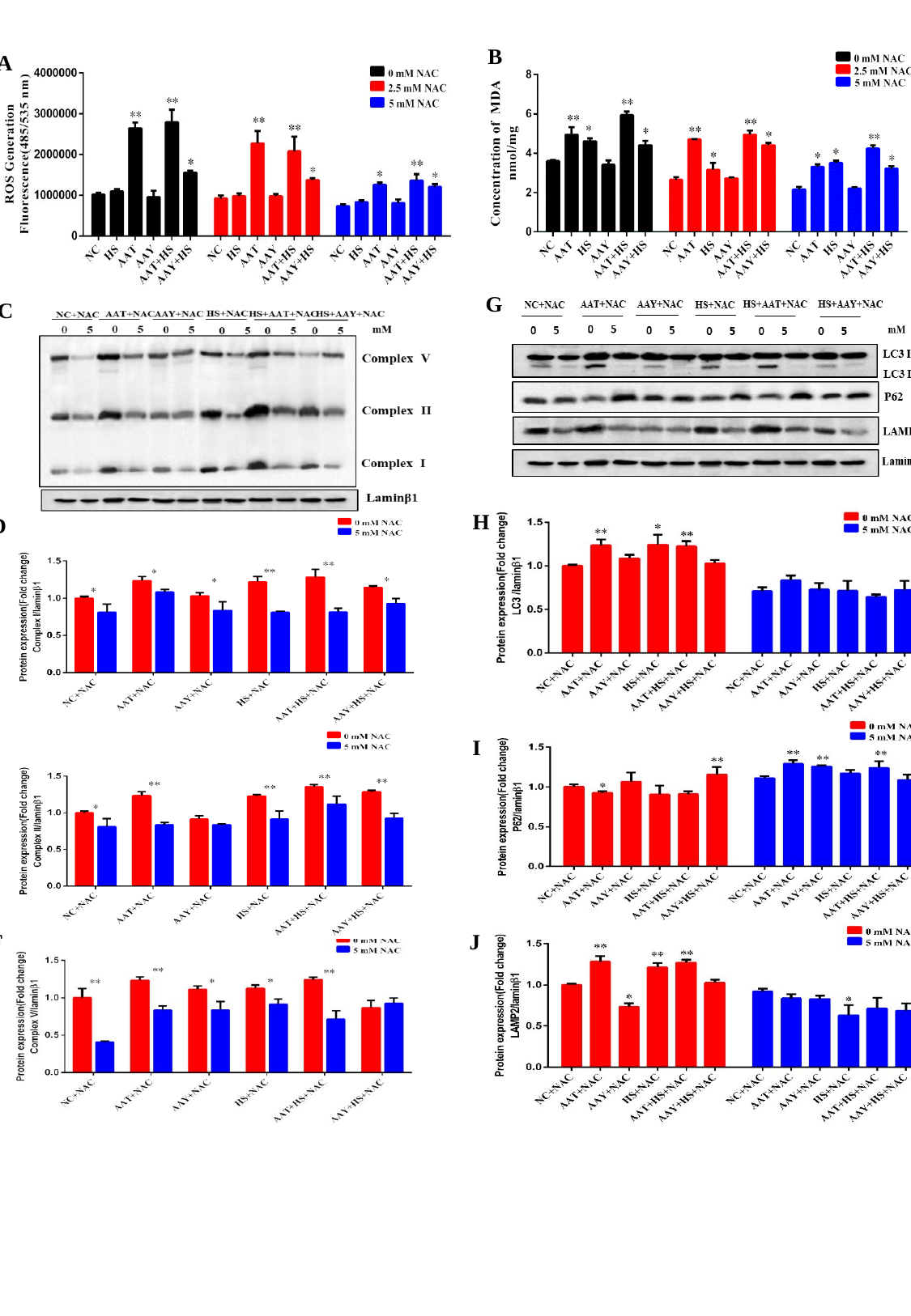

B
A
G
C
H
I
J
D
E
F

## Slide 7
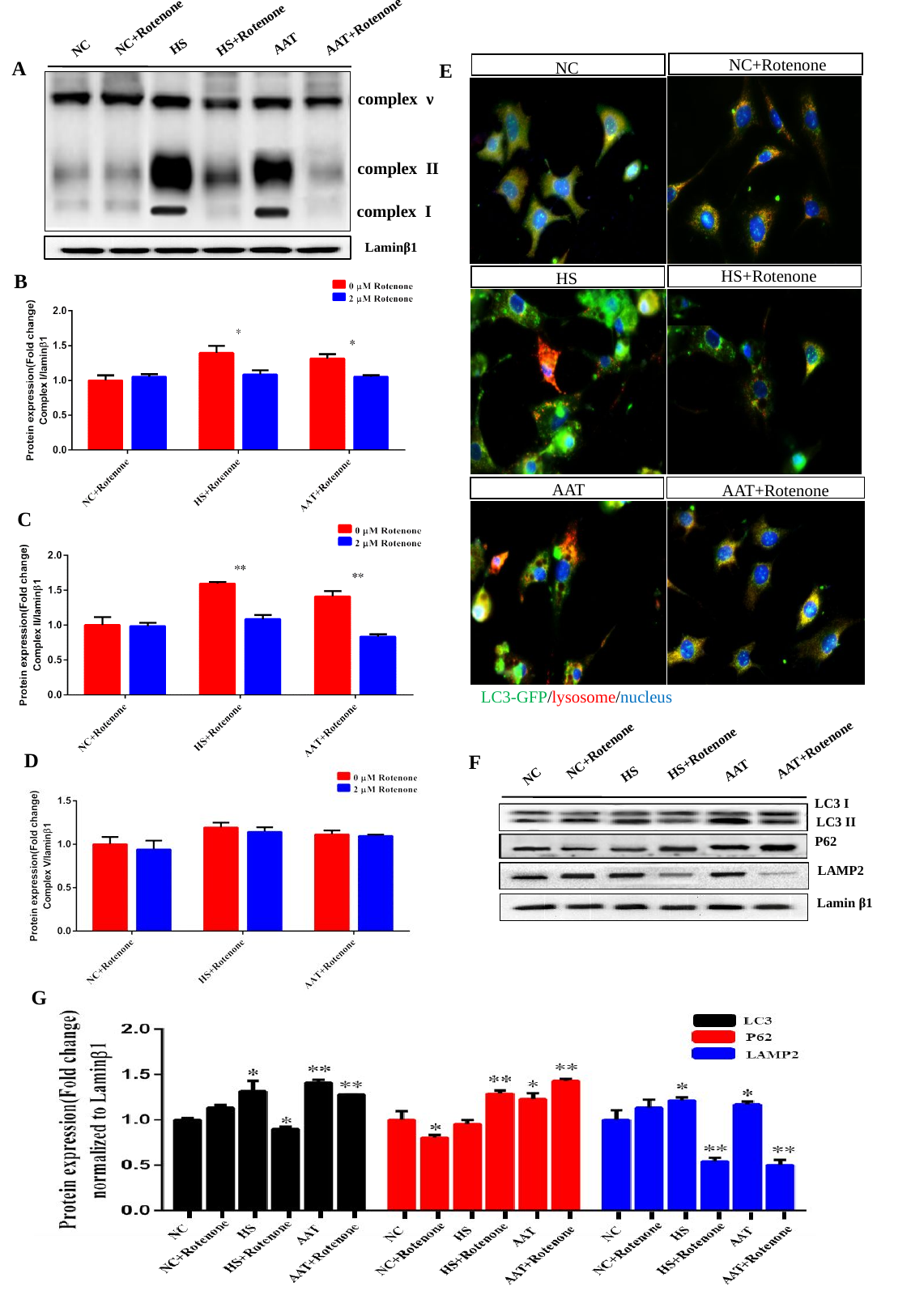

AAT+Rotenone
NC+Rotenone
HS+Rotenone
AAT
HS
NC
complex ν
complex II
complex I
Laminβ1
A
NC+Rotenone
NC
 HS+Rotenone
HS
AAT
AAT+Rotenone
LC3-GFP/lysosome/nucleus
E
B
C
AAT+Rotenone
NC+Rotenone
HS+Rotenone
AAT
HS
NC
LC3 I
LC3 II
P62
LAMP2
Lamin β1
F
D
G

## Slide 8
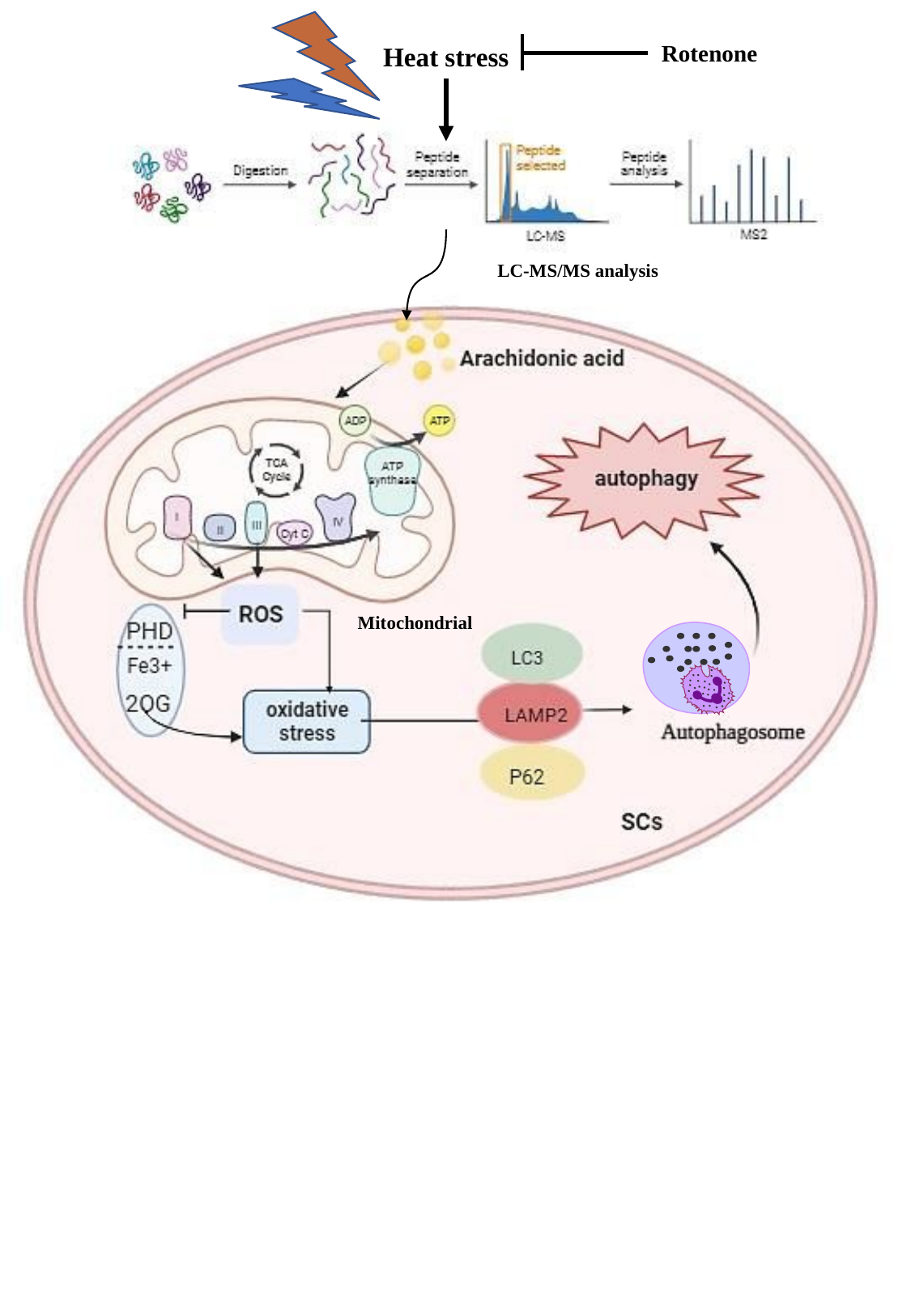

Heat stress
LC-MS/MS analysis
Mitochondrial
Rotenone
