# Supplementary material for: Heat stress upregulates arachidonic acid to trigger autophagy in sertoli cells via dysfunctional mitochondrial respiratory chain function
Source: J Transl Med. 2024 May 26;22:501. doi: 10.1186/s12967-024-05182-y (PMC11129461; doi:10.1186/s12967-024-05182-y)
Supplement: Supplementary file 4 — Supplementary Material 4 [file 12967_2024_5182_MOESM4_ESM.docx]

**Point by point responses to reviewer**

We would like to express our thanks sincerely for your kind interest in our manuscript entitled “Heat stress releases arachidonic acid to induce autophagy in Sertoli cells via dysfunctional mitochondrial respiratory chain function” (JTRM-D-23-10815R1) written by Hu et al. We are grateful to the esteemed reviewers for their critical and constructive comments which significantly improved our manuscript. We have accepted the reviewer' suggestions and modified or justified all the points after receiving and reading your comments. All changes made to the text are in red color so that they may be easily viewed.

**Reviewer #2**

**1.Question:** The article has been substantially revised and improved. Consider using instead of "arachidonic acid inhibitor" a term like arachidonic acid metabolism inhibitor or arachidonic acid synthesis inhibitor, depending on the scope of your research. Furthermore, a reference shall be added about effect of the specific compound as arachidonic acid synthesis inhibitor in mammalian cells.

***Answer: Thank for your suggestions. Diethylcarbamazine citrate is arachidonic acid metabolism inhibitor and we replaced arachidonic acid metabolism inhibitor, instead of "arachidonic acid inhibitor. In our study, our objective is to provide proof enough to show the effect of arachidonic acid. Thus, we used an arachidonic acid metabolism inhibitor (Diethylcarbamazine citrate). Mounting evidence showed that PPOH is an inhibitor of CYP epoxidase activity on arachadonic acid, which belongs to arachidonic acid synthesis inhibitor. The suggestion on arachidonic acid synthesis inhibitor in mammalian cells is helpful to determine the effect of arachidonic acid, because PPOH showed anti-inflammatory activity in endothelial cells (Reference : Ara A Askari, Scott Thomson, Matthew L Edin, Fred B Lih, Darryl C Zeldin, David Bishop-Bailey. Basal and inducible anti-inflammatory epoxygenase activity in endothelial cells. Biochem Biophys Res Commun. 2014,446(2):633-7). In the future, we will do some study on effect of PPOH in Sertoli cells.***

2.**Question:** Meanwhile, with the increase of the treatment. Line 269 time (0 to 6 h) for the 100 µM AAT and HS (0 to 1h), cell viability declined compared to the control, whereas the cell viability increased in the AAY (P < 0.01, Fig.2B).AAY treated cell viability increases and decreases relative to NC, so it is not clear wat it means? HS is observed for short segment, why? HS does not show extreme fall in cell viability.

***Answer:*** ***The Methods and Materials stated that SCs HS processed for 1 h, and since AA and HS are two different treatments, and the different time .The effect of heat stress depended on time and intensity. Long-term heat treatment will result in cell death, which is not the state we wish to detect.***

**3.Question:** Line 289 0.5-fold increase - is it meant increase by 50%? Or decrease?

***Answer:*** ***Thanks for your comments, is it meant increase by 50% or increase 1.5-fold.We have corrected the relevant value. Line 290***

**4.Question:** Line 383 what is exactly meant by electric leak?

***Answer: Superoxide production, such as ROS and MDA, is the result of electron leakage from the mitochondrial respiratory chain. A leak of electrons occurs when electrons exit before reducing oxygen to water at complex IV (cytochrome c oxidase) protein functions, and they react instead with oxygen to produce superoxide.***

**5.Question:** Line 242 HS upregulated AA level in SCs Please, add exact information what quantitively the increase in AA was?? Relatively, and in comparison to NC.

***Answer:*** ***In heat stressed SCs, AA was upregulated (about 2.35-fold) compared to NC. The details have been added in the text. Line 258***

**6.Question:** Line 259 Suggesting that AA plays an important role during HS, and may exhibit a switch-like response in multiple metabolic pathways during HS. This is of suggestive nature. Therefore conclusions must also emphasize suggestive nature of your exploratory research.

***Answer: Thank for your advice. We have revised it in new version. Line 259-261. Line39. Line 443***

**7.Question:** Line 344 Both HS and AA elevated ROS and MDA concentrations in cells. Are they concurrent or competitive processes?

***Answer: The two processes are concurrent. Overproduction of reactive oxygen species (ROS) can lead to lipid peroxidation, which is often measured by measuring malondialdehyde (MDA) levels in response to HS and AA stimulations.( Reference: Reactive oxygen species (ROS), Total antioxidant capacity (AOC) and Malondialdehyde (MDA) make a triangle in evaluation of Zinc stress extension)***
